# Identification of an Osteochondral Erosion Process in Gout Driven by Fibrinogen‐Integrin Activation and Local MSU Crystallization

**DOI:** 10.1002/advs.76383

**Published:** 2026-07-13

**Authors:** Hanlin Xu, Simin Bi, Zhechen Liu, Xiaofeng Zhou, Hengwei Qin, Nicola Dalbeth, Minbiao Ji, Yinghui Hua

**Affiliations:** ^1^ Department of Sports Medicine Huashan Hospital Fudan University Shanghai China; ^2^ Department of Radiology Huadong Hospital Shanghai Key Laboratory of Clinical Geriatric Medicine Shanghai Institute of Geriatrics and Gerontology State Key Laboratory of Surface Physics and Department of Physics Fudan University Shanghai China; ^3^ Department of Medicine The University of Auckland Auckland New Zealand

**Keywords:** Cartilage erosion, Fibrinogen, Gout, Integrin signaling, MSU crystallization

## Abstract

Gout is characterized by monosodium urate (MSU) crystal deposition, recurrent inflammatory flares, and progressive cartilage erosion. However, the reasons for preferential MSU crystal formation at cartilage injury sites and the drivers of cartilage degradation remain unknown. Here, the dynamics of MSU crystal deposition in cartilage were investigated using stimulated Raman scattering microscopy combined with isotope labeling. MSU crystals preferentially accumulated at cartilage injury sites in a crystallization‑frequency‑dependent pattern, and multicycle MSU crystallization promoted deeper deposition in injured cartilage. Proteomics revealed local enrichment of the fibrinogen complex following cartilage injury. In vivo and in vitro validation experiments further showed that fibrinogen gamma chain (FGG) deposition co‑localized with local MSU accumulation, accelerated MSU crystallization, and determined the site preference of deposition in a gout model. FGG and MSU deposition were further linked to integrin‑related adhesion signaling and matrix‐degrading programs. Cartilage injury, FGG deposition, and selective MSU crystallization together constitute a vicious cycle in gouty cartilage erosion, which can be interrupted by urokinase treatment or cartilage barrier restoration. In conclusion, cartilage injury promotes local fibrinogen deposition and deeper crystal invasion, thereby forming a self‑amplifying loop that contributes to gout‑associated cartilage erosion. This study also provides new biomarkers and therapeutic targets for gout management.

## Graphical abstract

Cartilage injury promotes local fibrinogen deposition, which accelerates monosodium urate crystallization and activates integrin‐mediated matrix‐degradation. This self‐amplifying cycle drives gout‐related cartilage erosion. Disrupting fibrinogen deposition or restoring the cartilage barrier could interrupt this vicious cycle.

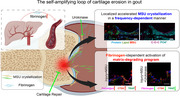

## 1 Introduction

Gout is a common type of inflammatory arthritis characterized by the deposition of monosodium urate (MSU) crystals within the joints, leading to flares, chronic arthritis, and cartilage erosion [1]. When the disease progresses to tophaceous gout, joint deformity and disability may arise from osteochondral injury [2]. According to studies, MSU crystal deposition and cartilage erosion exhibit strong site specificity, showing a preference for joints affected by osteoarthritis (OA) or early cartilage injury [3, 4]. This pronounced site‐specific preference underscores the importance of intra‐articular risk factors in influencing MSU deposition and gout pathogenesis [5].

Among these factors, injured cartilage itself is considered an important mediator of the intra‐articular microenvironment in multiple ways, thereby creating conditions that favor MSU crystal formation and gout flares [6, 7]. It could increase glycolytic activity and lactate accumulation and cause local acidification, thereby influencing urate solubility and MSU crystallization [8, 9]. Cartilage injury also causes broader changes in locally recruited or deposited protein components. For example, type II collagen released from injured cartilage can change MSU crystal morphology and promote crystal‐induced inflammatory responses [7]. The deposition of immune‐ and coagulation‐related proteins at cartilage injury sites can also bind MSU crystals and modulate crystal‐mediated inflammation [10, 11]. However, despite substantial evidence, these studies are largely observational, focusing on how isolated factors affect MSU crystallization or crystal‐induced inflammatory responses [6]. The lack of in situ joint observations has made it difficult to integrate all these factors to identify the primary drivers of the process, as well as to determine the interactions among MSU crystallization, joint tissue, and these driving factors.

Cartilage explant models represent a reliable and innovative modality for addressing these gaps. Cartilage explants are living, functional organ portions that bridge the gap between traditional two‐dimensional (2D) cell cultures and animal models, replicating the composition and structure of joint tissues with high fidelity [12]. Compared with 2D cell models (which lack the complexity of joint tissue components and yield unreliable biological responses), cartilage explant models maintain cell viability for 2–3 weeks, providing an adequate time frame for in vitro studies [12, 13]. The integration of advanced biophotonics techniques, including Raman spectroscopy, and stimulated Raman scattering (SRS) microscopy, enables noninvasive molecular characterization and high‐resolution chemical imaging, making these methods suitable for analyzing the structure and composition of cartilage [14, 15]. When integrated with cartilage explant models, SRS microscopy enables high‐resolution in situ mapping of cartilage structure, visualization of localized MSU crystal distribution, and monitoring of changes in protein‐associated signals within the cartilage matrix [16]. Stable isotope labeling enhances these studies by enabling the precise tracking of deposition dynamics and facilitating detailed in situ cartilage analysis [17, 18].

In the present study, time‐course and multicycle MSU crystallization assays were combined with isotope labeling to define MSU crystallization patterns on intact or injured cartilage surfaces. Furthermore, mass‐spectrometry was used to examine the most significant changes in the local protein environment associated with cartilage injury and MSU crystal deposition, and experimental validation was performed to explore the links between the identified proteins and the rate and location of crystal formation. Mouse and human data were further integrated to assess the effects of cartilage‐injury‐associated protein changes and MSU deposition on signaling pathways and cell activation in injured cartilage regions. The primary objective of the study was to define the interactions among the dynamic changes and preferential localization of MSU crystals, alterations in the protein environment, and cartilage erosion within the joint system.

## 2 Results

### 2.1 Raman Spectroscopy and SRS Reveal the Effects of MSU Crystals on Cartilage Explant Microstructure

**FIGURE 1:**
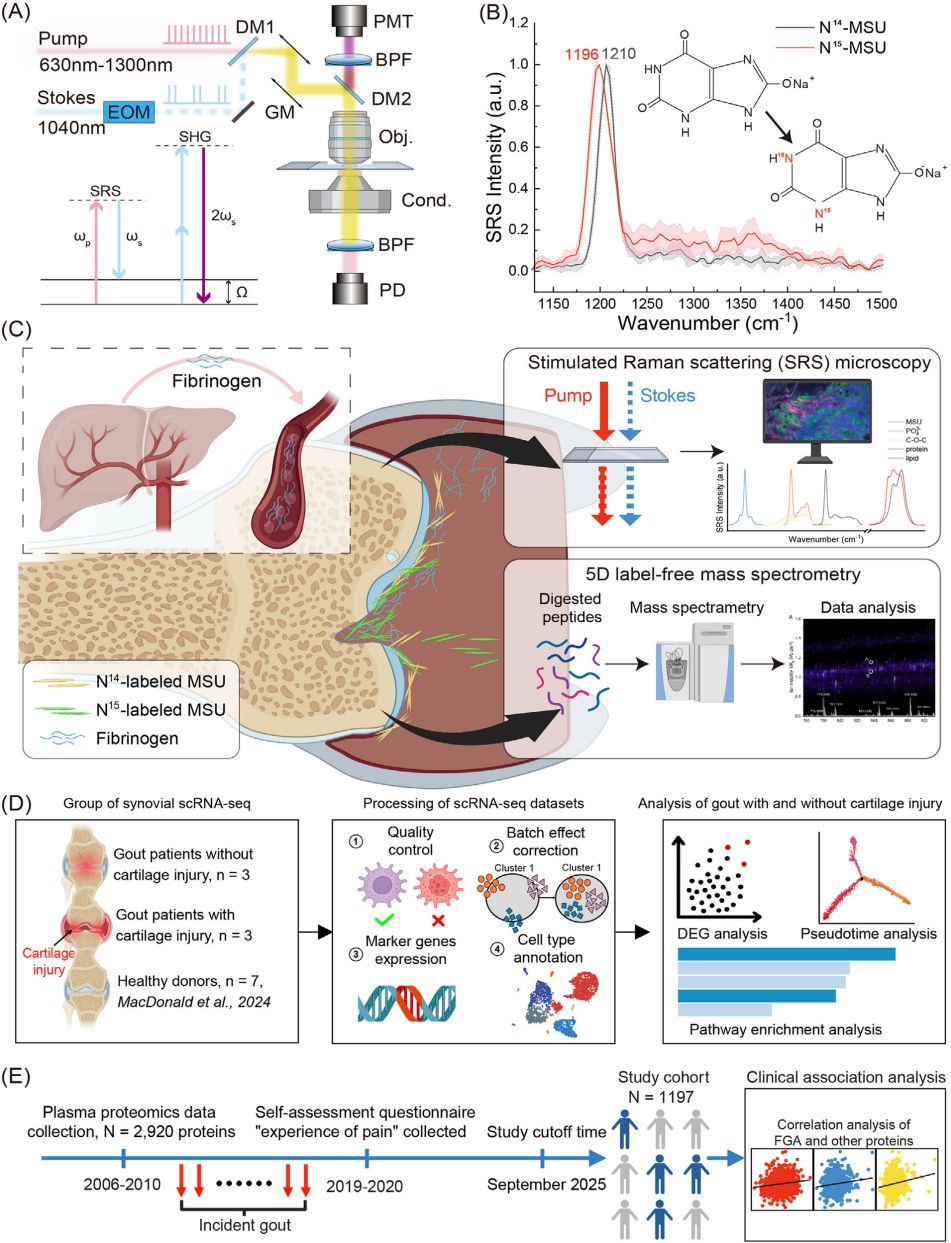
Schematic of the experimental approach. (A) Principle of SRS microscopy and the beam path diagram. (B) C‐^14^N and C‐^15^N bonds produced SRS spectra at distinct peak positions. (C) SRS and 5D mass spectrometry were used to elucidate the role of liver‐derived fibrinogen in secondary MSU crystallization and cartilage erosion. (D) Workflow of single‐cell RNA‐seq data integration and analysis. (E) Flowchart of participant selection and analysis in the UK Biobank (UKB) study.

**FIGURE 2:**
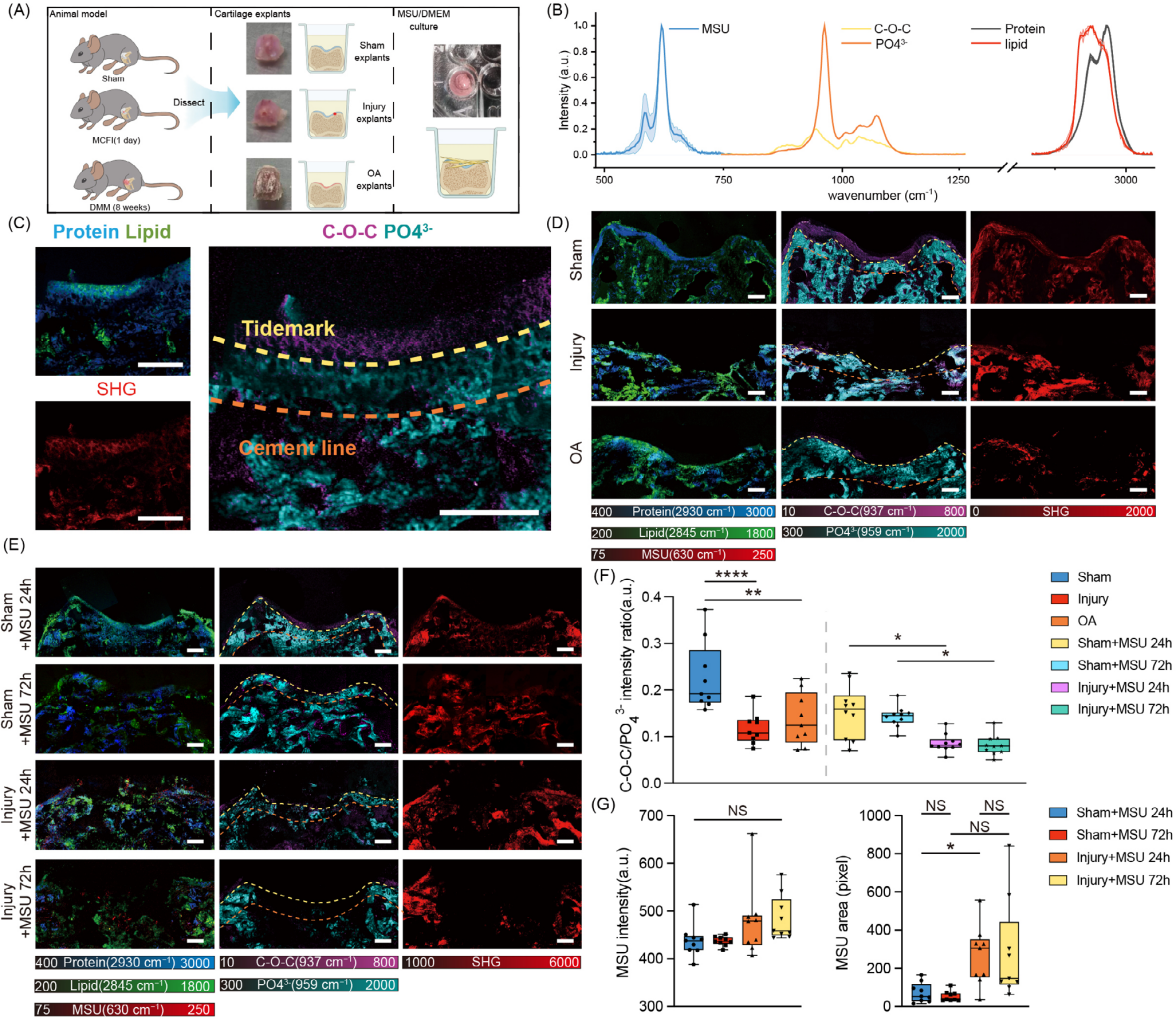
SRS microscopy reveals the effect of MSU crystals on cartilage tissue microstructures. (A) Schematic representation of cartilage explants subjected to various treatments (*n* = 5 per group), co‐cultured with either saturated MSU in DMEM or DMEM alone as a control for subsequent observation. (B) SRS spectra showing characteristic vibrational peaks associated with MSU, bone, and cartilage molecular bonds. (C) SRS images showing microstructural features of the cartilage and bone, with dashed lines (Yellow, orange, indicating the tidemark, cement line, respectively) indicating boundaries between the noncalcified and calcified zones of the cartilage and subchondral bone. (D) Representative SRS images from the sham, injury, and OA groups across different SRS channels, with signal ranges standardized using a look‐up table. Yellow dashed lines, orange dashed lines, indicate the tidemark, cement line, respectively. (E) Representative SRS images from the sham and injury groups co‐cultured with MSU for 24 or 72 h, with signal ranges standardized using a look‐up table. Yellow dashed lines, orange dashed lines, indicate the tidemark, cement line, respectively. (F,G) Quantitative analysis, normalized to the signal intensity at 959 cm^−1^, showing the C─O─C/PO_4_^3−^ intensity ratio, along with MSU deposition intensity (G, left) and area (G, right) on the cartilage surface. For quantitative analysis, three mice were included per group (*n* = 3), and 2–3 representative fields of view were analyzed for each mouse. MCFI, medial condylar femoral injury model; DMM, destabilization of the medial meniscus; Scale bars: 100 µm. NS, not significant; ^*^ *p* < 0.05, ^**^ *p* < 0.01, ^***^ *p* < 0.001, ^****^ *p* < 0.0001.

Multimodal imaging of knee cartilage explants was performed using SRS and second harmonic generation (SHG) microscopy to investigate the effects of MSU on the microstructure and molecular composition of mice cartilage explants (Figure 1). SRS‐imaged and non‐imaged control sections were compared to exclude the potential effects of the imaging procedure on cartilage tissue. No significant difference was observed between the two groups (*p* > 0.05; Figure S1A–C). To simulate in vivo MSU crystal formation, urate‐saturated Dulbecco's Modified Eagle Medium (DMEM) was prepared and applied to Sham, injured, or OA explants obtained from mice (*n* = 5 per group) (Figure 2A). SRS spectroscopy identified characteristic vibrational peaks of MSU, phosphate, C─O─C groups, proteins, and lipids at 630, 959, 937, 2930, and 2845 cm^−1^, respectively, which were consistent with those reported previously (Figure 2B) [16, 19].

SRS spectroscopy of various characteristic peaks imaged cartilage and bone tissue microstructures, including the tidemark (yellow, dash line) and cement line (orange, dash line) highlighted in Figure 2C. For injured samples, a boundary‐identification algorithm based on region‐specific tissue signals was further established to assist with the assignment of tidemark and cement line positions (Figure S2). These boundaries were further annotated in Figure 2C–E and Figure S3.

An observation model was established by collecting baseline SRS intensity data from the explants (Figure 2D). In the sham group, the protein and lipid signals remained stable, and C─O─C and PO_4_^3−^ signals were normally distributed in healthy cartilage. According to the SHG signals, the collagen fibers were well‐organized in the tissue. By contrast, in the OA and injury groups, the protein and C─O─C signal intensities in the noncalcified cartilage zone were decreased or absent, whereas the lipid signal intensity increased. This finding suggests a loss of noncalcified cartilage and surface collagen disruption.

Subsequently, the time‐dependent accumulation of MSU crystals was investigated for over 24–72 h (spanning the peak inflammatory phase of most gout flares). The findings demonstrated that the integrity of noncalcified cartilage protects deeper tissues from erosion by MSU. After 24 h of co‐culture, the cartilage in the sham group showed intact signals across all channels, no MSU crystal deposition on the surface, and largely preserved collagen fibers in SHG imaging. By contrast, the cartilage showed MSU crystal deposition on the surface at 24 and 72 h and structural damage in the injury group (Figure 2E). Based on these findings, the injured cartilage surface provides a more favorable microenvironment for local MSU crystal deposition.

The PO_4_^3−^ signal at 959 cm^−1^ from the subchondral bone plate, measured in the area delineated by white dashed boxes, was used as an internal reference to normalize the C─O─C signal because the PO_4_^3−^ signal intensity in bone trabeculae within the same batch of samples remained stable after cartilage injury and MSU treatment (*p* > 0.05, Figure S3). Therefore, the C─O─C/PO_4_^3−^ intensity ratio was used as the normalized C─O─C signal. Quantitative analysis showed that the normalized C─O─C signal intensity was significantly higher in the sham group than in the injury (*p* < 0.0001) and OA groups (*p* < 0.01) (Figure 2F). This finding supports that the injury and OA groups had loss of noncalcified cartilage matrix compared with the sham group, and the calcified signals in the calcified cartilage zone and subchondral bone plate remained relatively stable.

After 24 and 72 h of MSU crystallization, the normalized C─O─C signal intensity remained lower in the injury+MSU group than in the sham+MSU group (24 h, *p* < 0.05; 72 h, *p* < 0.05; Figure 2F). Moreover, MSU crystallization significantly reduced the normalized C─O─C signal intensity in the sham (24 h, *p* < 0.05; 72 h, *p* < 0.01; no significant difference between 24 and 72 h) and injury groups (24 h, *p* < 0.05; 72 h, *p* < 0.05; no significant difference between 24 and 72 h) compared with the corresponding baseline condition without MSU crystallization (Figure S4A,B). Quantification of MSU deposition showed no significant difference in MSU signal intensity between the injury and sham groups (Figure 2G, left). However, the MSU deposition area on the cartilage surface was significantly larger in the injury group after 24 h of treatment (*p* < 0.05; Figure 2G, right). Taken together, the time‐course MSU crystallization assay showed that initial cartilage injury was associated with reduced noncalcified cartilage matrix signals and increased MSU surface deposition area. However, these changes did not show further time‐dependent progression under a single MSU crystallization condition (24 vs. 72 h; Figure 2F,G).

### 2.2 Isotope‐Labeled SRS Analysis Reveals the Effects of Multicycle MSU Crystallization on Cartilage Erosion

**FIGURE 3:**
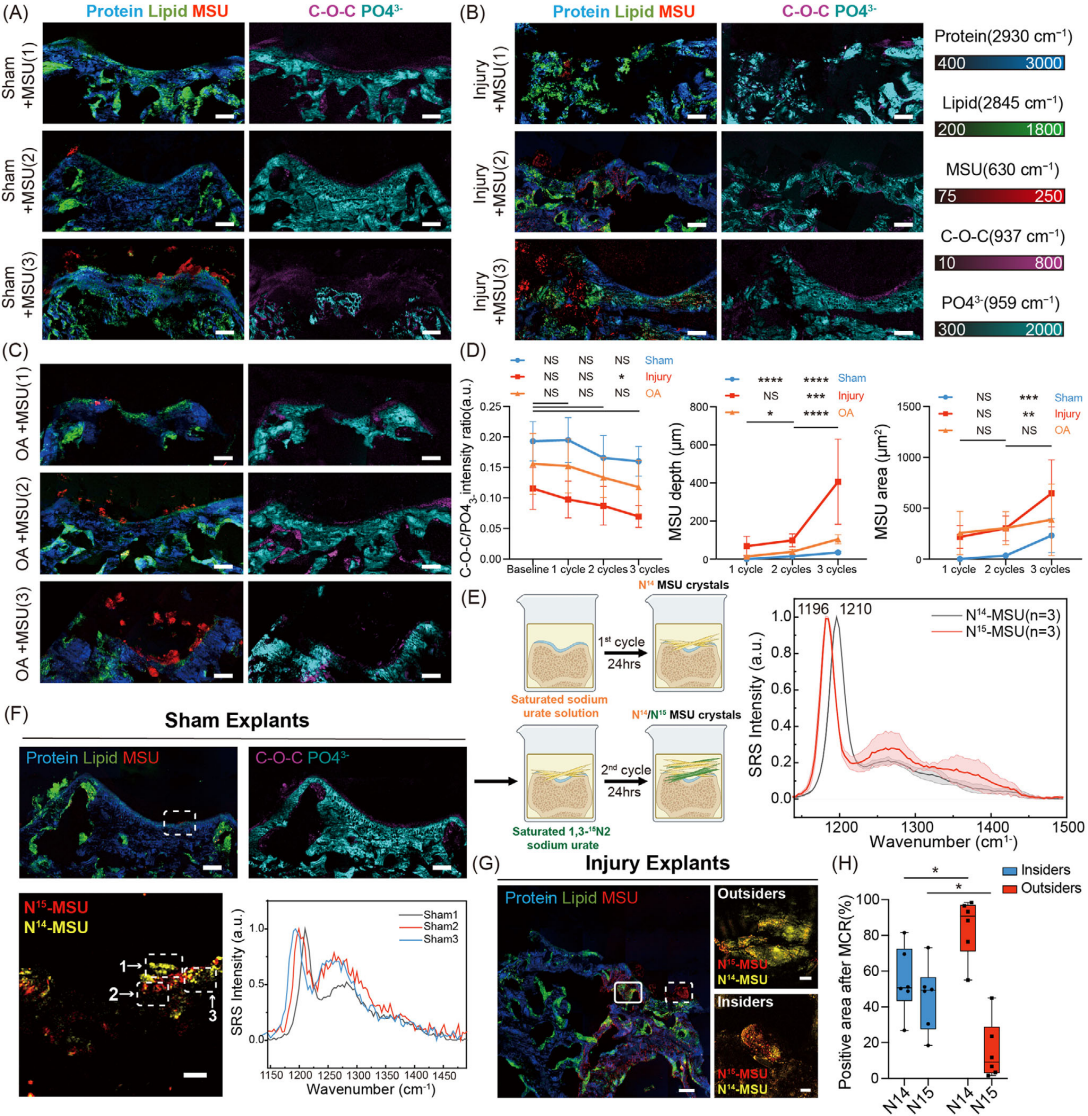
Isotope‐labeled SRS analysis reveals the effects of multicycle MSU crystallization on cartilage erosion. (A–C) Representative images from the sham, injury, and OA groups after MSU crystallization for 1–3 cycles across different SRS channels, with signal ranges standardized using a look‐up table (*n* = 5 per group). (D) Quantification of matrix changes and MSU deposition. Left: C─O─C/PO_4_^3^^−^ intensity ratio. Right: MSU deposition depth and area. For quantitative analysis, 21 representative fields of view were analyzed for each group. (E) Left: Schematic of cartilage explants subjected to sequential crystallization cycles using ^14^N‐ or ^1^ ^5^N‐labeled saturated sodium urate in DMEM for subsequent analysis. This approach distinguishes MSU crystals formed during different crystallization cycles. Right: Isotope discrimination based on the Raman peak near 1200 cm^−^^1^, corresponding to the C─N stretching mode, with ^1^ ^5^N‐labeled MSU showing a distinct redshift in this region. (F) Isotope‐labeled MSU crystals in cartilage explants, with little interference from tissue background near 1200 cm^−^^1^, enabling isotope‐based crystal differentiation. Dashed boxes indicate representative MSU‐enriched regions selected for hyperspectral SRS analysis. (G) Representative SRS images of outer and inner MSU crystal regions in injured cartilage explants. Left: Spatial distribution of MSU crystals. Solid boxes indicate “Insiders”; dashed boxes indicate “Outsiders.” Right: Multivariate curve resolution analysis showing ^1^ ^5^N‐labeled (red) and ^14^N‐labeled (yellow) MSU crystals. (H) Quantification of ^14^N‐ and ^1^ ^5^N‐positive areas in “Insiders” and “Outsiders” regions following MCR analysis. For quantitative analysis, six representative fields of view were analyzed for both “Insiders” and “Outsiders.” Scale bars: 100 µm; magnified images in (F,G): 20 µm. NS, not significant; ^*^ *p* < 0.05, ^**^ *p* < 0.01, ^***^ *p* < 0.001, ^****^ *p* < 0.0001.

Given that MSU deposition on the cartilage surface does not progress over time and that recurrent gout flares are associated with cartilage erosion in patients with gout [20, 21], whether multicycle MSU crystallization affected the depth of crystal deposition and subsequent cartilage erosion was further examined. In the sham group, the normalized C─O─C signal intensity remained relatively high and did not significantly decrease with increasing crystallization cycles. By contrast, the injury group showed lower normalized C─O─C signal intensity than the sham group after each cycle (one cycle, *p* < 0.001; two cycles, *p* < 0.01; three cycles, *p* < 0.0001; Figure 3A–D and Figure S5). In the sham group, although the MSU signals gradually increased on the cartilage surface, the intact noncalcified cartilage layer still limited MSU invasion into deeper tissues. Moreover, MSU deposition remained restricted to superficial regions even after three crystallization cycles, with low deposition depth (mean: 13.77 µm) and deposition area (mean: 255.27 µm^2^) (Figure 3D, right). In the injury group, increasing crystallization cycles were accompanied by marked disruption of the SHG collagen signal and a further decrease in normalized C─O─C signal intensity (three cycles vs. baseline, *p* < 0.05; Figure 3D, left; Figure S6). Furthermore, the MSU crystal depth (*p* < 0.001) and deposition area (*p* < 0.01) in the injury group reached their highest levels after three crystallization cycles (Figure 3D, right). These findings support that cartilage barrier disruption is required for multicycle MSU crystallization to develop into deeper erosion. The OA group was overall closer to the sham group in normalized C─O─C signal intensity, MSU deposition depth, and MSU deposition area and did not show the marked deep crystal invasion observed in the injury group (Figure 3B–D).

To further determine how each crystallization cycle contributed to deeper MSU crystal invasion into cartilage, nitrogen isotope labeling was used to distinguish MSU crystals that were formed during different crystallization cycles. ^14^N‐sodium urate was used for the first cycle, and 1,3‐^15^N_2_‐sodium urate was used for the second cycle (Figure 3E, left). The Raman peak near 1200 cm^−1^, corresponding to the C─N stretching mode, was selected for isotope differentiation because ^15^N‐labeled MSU crystals showed a clear redshift near 1200 cm^−1^ and this region had little interference from the tissue background [22] (Figure 3E, right; Figures S7 and S8A–C). Subsequently, hyperspectral SRS images around 1200 cm^−1^ were acquired in a designated small field of view enriched with MSU signals using a higher‐magnification objective (Figure 3F, white dotted rectangle). The hyperspectral signals were further unmixed using the multivariate curve resolution (MCR) algorithm to reconstruct the spatial distributions of ^14^N‐ and ^15^N‐labeled MSU crystals within cartilage tissue, which had characteristic Raman peaks at 1210 and 1196 cm^−1^, respectively. In addition, Raman spectra from representative sampling points showed differences in isotope composition and signal intensity across different positions, reflecting clear spatial heterogeneity in MSU deposition (Figure 3F, lower).

Based on their position relative to the tidemark, the cartilage regions that MSU crystals deposited were divided into “Outsiders” and “Insiders”. Subsequently, SRS imaging and MCR‐based spectral unmixing were performed separately for these two spatial regions (Figure 3G, left). The “Outsiders” and “Insiders” regions contained detectable ^14^N‐ and ^15^N‐labeled MSU signals (Figure 3G, right). However, the key difference was that the “Insiders” regions showed stronger enrichment of ^15^N‐labeled MSU signals, whereas ^14^N‐labeled signals were more scattered and did not show a comparable level of enrichment in deeper regions (Figure 3G, right). This finding shows that multicycle MSU crystallization can produce spatially distinct MSU deposition patterns within injured cartilage. MCR‐based quantitative analysis showed that the ^15^N‐positive area was significantly higher in the “Insiders” than in the “Outsiders” regions (*p* < 0.05). This finding indicates that the MSU crystals formed during the second crystallization cycle preferentially accumulated in deeper regions of injured cartilage (Figure 3H). Together, these results demonstrate that multicycle MSU crystallization, especially a subsequent crystallization event within a short interval, promotes MSU crystal deposition in injured cartilage, with later crystallization cycles preferentially depositing at deeper cartilage erosion sites.

### 2.3 Proteomics Reveals the Enrichment of the Fibrinogen Complex in Injured Cartilage

**FIGURE 4:**
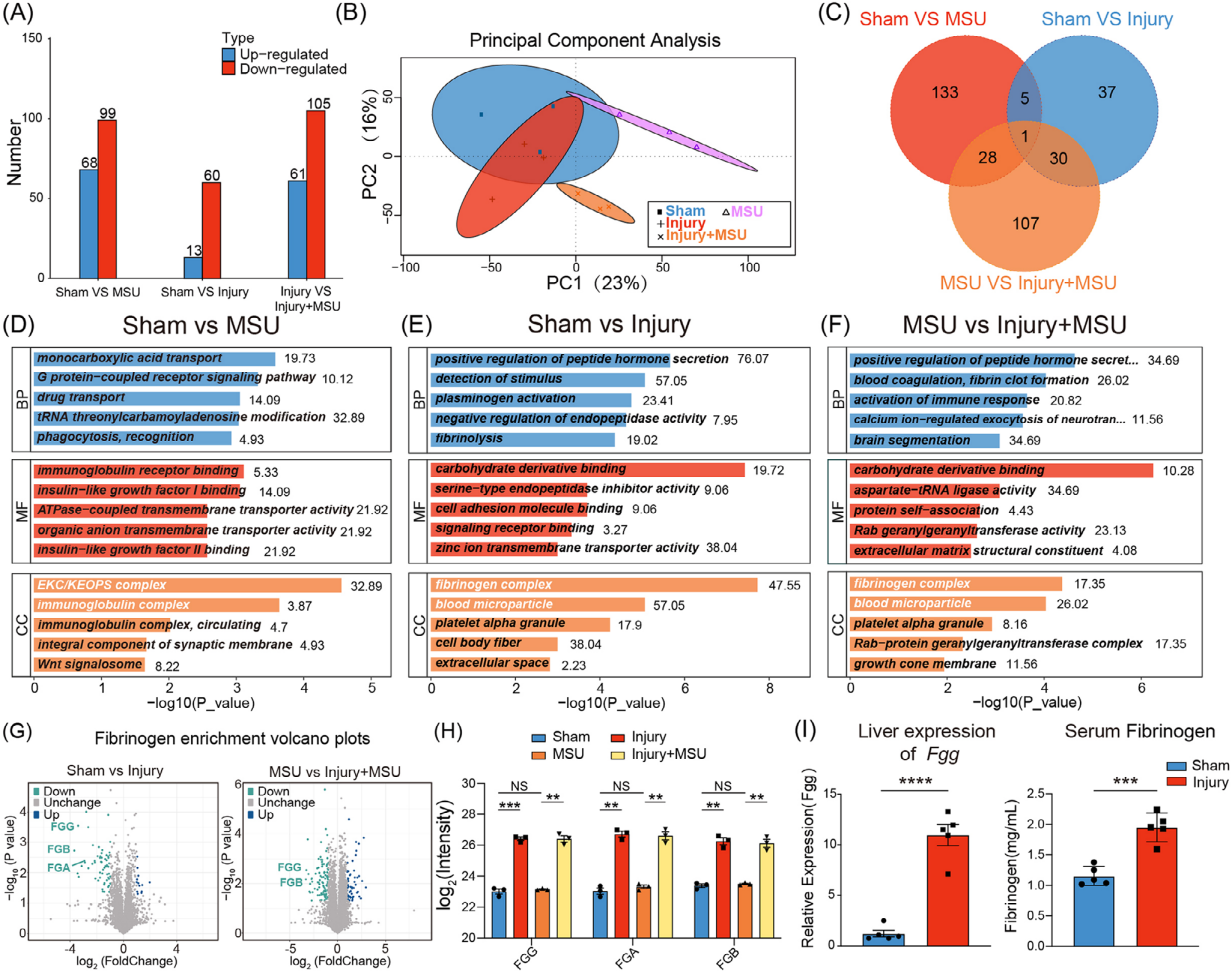
Urate‐induced alterations in cartilage proteomics following MSU crystal‐mediated cartilage erosion. (A) Label‐free 5D mass spectrometry showing upregulated or downregulated proteins across the groups (*n* = 3 per group). (B) PCA showing overall differences in protein expression across the groups. (C) Venn diagram comparing protein expression between the groups. (D–F) GO functional enrichment analysis of three comparison pairs: sham vs. MSU, sham vs. injury, and MSU vs. injury+MSU. Enrichment was performed across the three major GO domains: biological process (BP), molecular function (MF), and cellular component (CC). (G) Volcano plots showing the most significantly altered proteins in the sham vs. injury and MSU vs. injury+MSU comparisons. (H) Expression of fibrinogen chains (FGA, FGB, and FGG) across groups. (I) Liver Fgg mRNA expression and serum fibrinogen protein levels after cartilage injury (*n* = 5 per group). NS, not significant; ^*^ *p* < 0.05, ^**^ *p* < 0.01, ^***^ *p* < 0.001, ^****^ *p* < 0.0001.

Considering that spatially selective MSU deposition may be related to changes in the local or systemic protein milieu after cartilage injury, label‐free five‐dimensional (5D) proteomics was used to profile protein changes in cartilage explants from the sham group and from explants treated with injury and/or MSU. MSU treatment and cartilage injury altered the protein profiles of cartilage explants, with the most pronounced changes being observed under combined injury and MSU treatment (Figure 4A). Principal component analysis revealed overall differences in protein expression across groups (Figure 4B). In Figure 4C, the Venn diagram further showed overlapping and group‐specific proteins across different treatment groups (Table S1).

Gene Ontology functional enrichment analysis was further performed on differentially expressed proteins across groups, covering biological process, molecular function, and cellular component, with a focus on cartilage‐injury‐related changes in cellular component terms. The sham vs. MSU comparison mainly showed an enrichment of inflammation‐related protein terms, including immunoglobulin‐related components (Figure 4D). By contrast, the sham vs. injury and MSU vs. injury+MSU comparisons exhibited significant enrichment of fibrinogen complex and blood‐microparticle‐related terms (Figure 4E,F). Consistent with these enrichment results, the volcano plots showed that the main components of the fibrinogen complex, including fibrinogen alpha chain (FGA) protein, fibrinogen beta chain protein, and fibrinogen gamma chain (FGG) protein, were significantly increased in the sham vs. injury and MSU vs. injury+MSU comparisons (all *p* < 0.01, Figure 4G). By contrast, MSU treatment alone did not significantly change the abundance of these proteins (Figure 4H). These findings suggest that the fibrinogen complex is markedly enriched after cartilage injury and represents an important feature of the altered local protein milieu in injured cartilage.

The hepatic *Fgg* mRNA level and serum fibrinogen protein level were measured after injury to further validate the proteomic findings and determine whether FGG enrichment was associated with systemic fibrinogen elevation after cartilage injury. The hepatic *Fgg* mRNA level was significantly increased 1 day after injury (*p* < 0.0001; Figure 4I, left). Meanwhile, enzyme‐linked immunosorbent assay (ELISA) showed a significant increase in serum fibrinogen protein level in the injury group (*p* < 0.001; Figure 4I, right). These results show that cartilage injury was accompanied not only by local enrichment of the fibrinogen complex, but also by increased hepatic *Fgg* mRNA level and circulating fibrinogen protein level. Elevated circulating fibrinogen may further accumulate in injured cartilage regions and contribute to the altered local protein milieu after injury.

### 2.4 Injury‐Induced Fibrinogen Enrichment Promotes Local MSU Deposition

**FIGURE 5:**
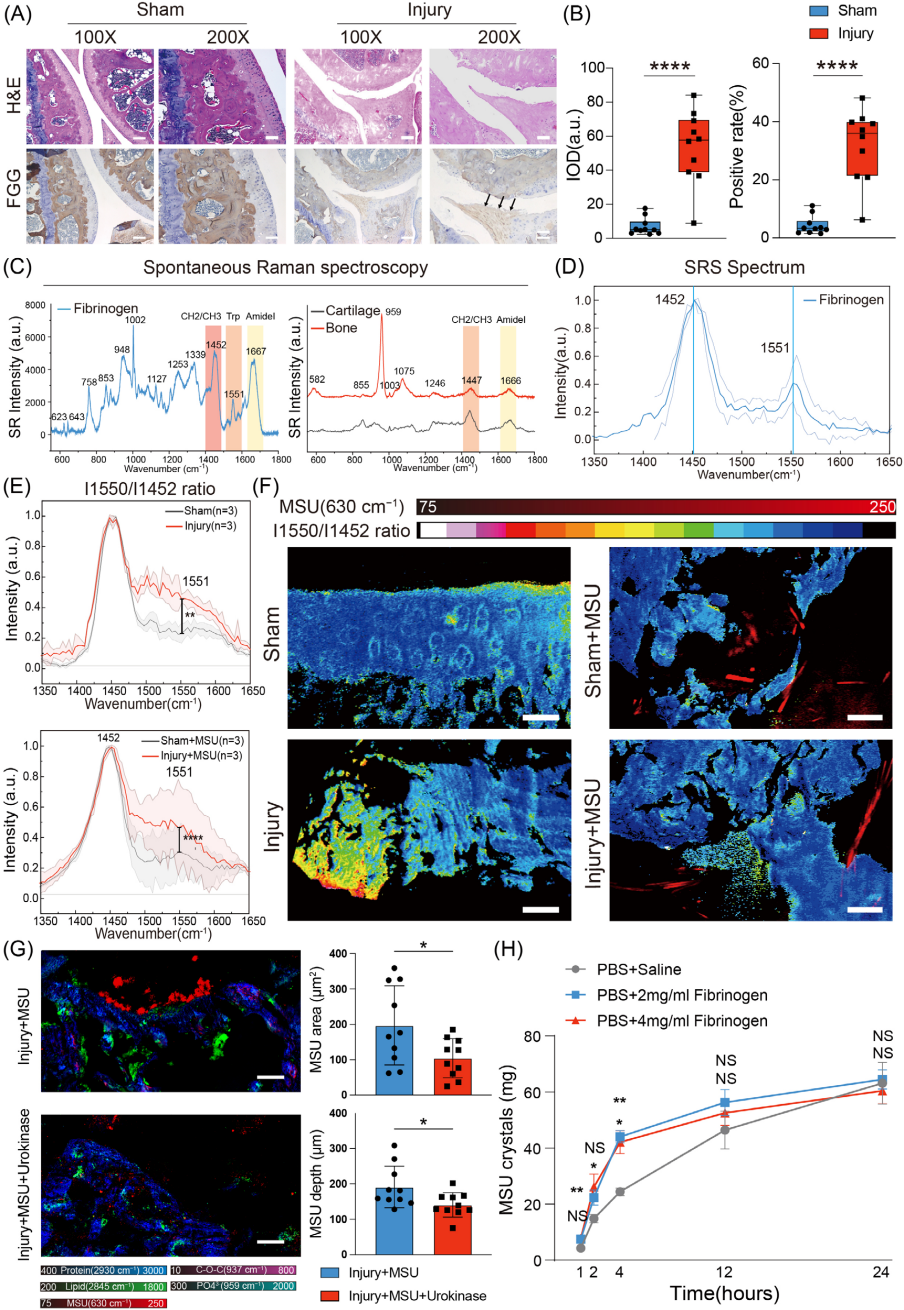
Injury‐induced fibrinogen enrichment promotes local MSU deposition in injured cartilage. (A) Histological staining revealing changes in the cartilage matrix following injury, including hematoxylin and eosin and FGG immunohistochemistry (*n* = 5 per group). (B) Quantification of FGG deposition by measuring integral optical density and the Fgg positive rate. For quantitative analysis, five mice were included per group (*n* = 5), and 3–4 representative fields of view were analyzed for each mouse. (C) Spontaneous Raman spectra demonstrate differences in fibrinogen deposition and the cartilage/bone background at the I_1452_/I_1551_ intensity ratio. (D) SRS spectra of pure fibrinogen around 1550 cm^−1^. (E) Representative Raman spectra of the cartilage tissue in the control and injury groups, with or without MSU deposition, showing changes in the I_1550_/I_1452_ ratio. (F) Left: Heatmap of the I_1550_/I_1452_ ratio revealing fibrinogen deposition in sham and injured cartilage. Right: Heatmap of the I_1550_/I_1452_ ratio combined with SRS images (red crystals) revealing a spatial correlation between fibrinogen and MSU crystal deposition in injured cartilage. (G) Representative SRS images and quantitative analysis of MSU deposition in injured cartilage with or without urokinase treatment. For quantitative analysis, five mice were included per group (*n* = 5), and two representative fields of view were analyzed for each mouse. (H) MSU crystallization rate curves following fibrinogen treatment at 2 and 4 mg/mL relative to the PBS+saline control (*n* = 5 per group). Scale bars: A, 50 µm; F, 20 µm; G, 100 µm. NS, not significant; ^*^ *p* < 0.05, ^**^ *p* < 0.01, ^***^ *p* < 0.001, ^****^ *p* < 0.0001.

We next sought to define the spatial distribution of FGG protein in injured cartilage and to examine the effect of increased FGG protein in injured cartilage on MSU deposition. Hematoxylin and eosin staining showed reduced chondrocyte density, disorganized cell arrangement, and local inflammatory cell infiltration in the injury group. Moreover, Alcian blue and Safranin O staining revealed weaker glycosaminoglycan staining in the injury group, consistent with proteoglycan loss and cartilage injury. In the injury group, FGG‐positive signals were clearly increased and mainly distributed at the cartilage surface and injury margins, as indicated by the arrows in Figure 5A. Quantitative analysis confirmed significant increases in the FGG‐positive area ratio and integrated optical density (IOD) of FGG signals in the injury group (both *p* < 0.0001), supporting increased local FGG protein deposition after cartilage injury (Figure 5B).

A characteristic Raman peak of purified fibrinogen was observed at 1550 cm^−1^ (Figure 5C,D). The I1550/I1452 ratio, which represents tryptophan‐related vibration relative to C─H bending vibration, was applied to characterize fibrinogen's spatial distribution. Quantitative analysis of SRS spectra supported the association between cartilage injury and increased fibrinogen‐related signals (Figure 5E). The I1550/I1452 ratio heatmap showed increased fibrinogen‐related signals at the injury margins and surrounding regions in the injury group, with stronger signals being observed in the deeper injured regions (Figure 5F, lower left). These signal‐enriched regions largely overlapped with MSU deposition areas in injured cartilage (Figure 5F, lower right). To test whether the enhanced MSU crystallization after cartilage injury is FGG‐dependent, we applied urokinase to eliminate FGG. Urokinase pretreatment reduced MSU deposition in injured cartilage regions, as shown by significant decreases in both MSU deposition area and invasion depth (both *p* < 0.05; Figure 5G). In vitro crystallization assay was also used to directly test the effect of fibrinogen on MSU crystal formation. Compared with the saline control, fibrinogen‐treated groups (2 and 4 mg/mL) showed higher MSU crystal mass at early time points (Figure 5H). Together, these results suggest that fibrinogen deposition may be an important driver of accelerated crystallization and selective deposition at sites of cartilage injury.

### 2.5 Fibrinogen Deposition Is Accompanied by the Activation of Integrin‐Mediated Matrix‐Degrading Programs

**FIGURE 6:**
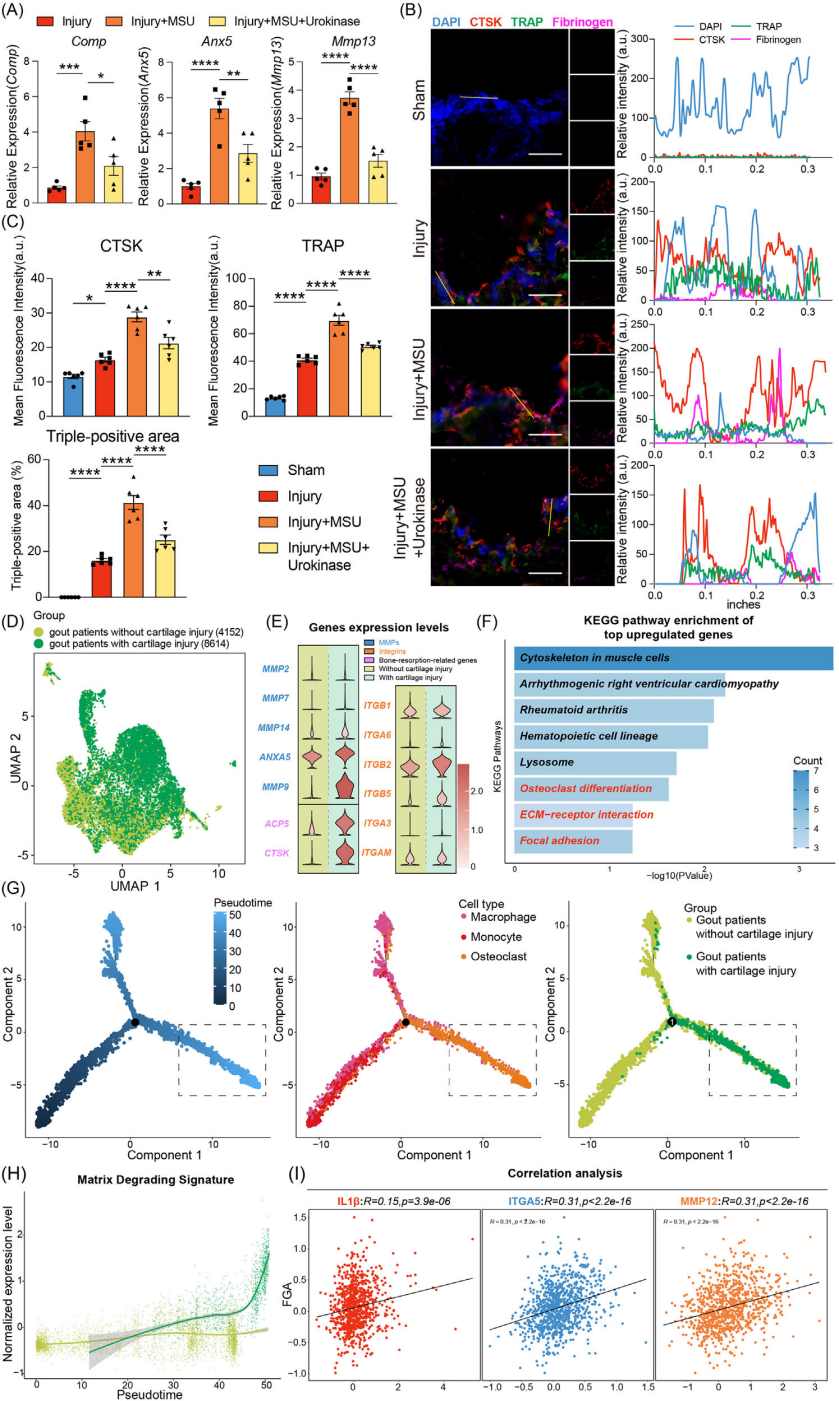
Fibrinogen deposition is associated with integrin‐mediated matrix‐degrading programs in gout. (A) *Comp*, *Anx5*, and *Mmp13* gene expression levels in cartilage across groups (*n* = 5 per group). (B) Representative immunofluorescence staining showing the spatial co‐localization of fibrinogen, tartrate‐resistant acid phosphatase (TRAP), and cathepsin K (CTSK) protein signals in injured mouse cartilage across groups. (C) Quantification of CTSK and TRAP mean fluorescence intensity and triple‐positive area across groups. (D) UMAP plots of integrated synovial mononuclear cell data visualized by clinical group. (E) Violin plots showing the expression levels of representative matrix‐degrading enzyme genes (blue), integrin family genes (orange), and bone‐resorption‐related genes (purple). (F) Kyoto Encyclopedia of Genes and Genomes (KEGG) pathway enrichment analysis of the top 80 upregulated genes in gout patients with cartilage injury. (G) Pseudotime trajectory analysis of synovial mononuclear phagocyte‐lineage cells from gout patients, visualized by pseudotime, cell type, and clinical group. Dashed boxes indicate the terminal differentiation branch. (H) Changes in the matrix‐degrading signature score along pseudotime. The signature score was calculated using a predefined matrix‐degradation‐associated gene set listed in Table S7. (I) Correlation analysis between plasma FGA protein levels and selected plasma proteins in gout patients from proteomic data. Scale bars: 50 µm. ^*^ *p* < 0.05, ^**^ *p* < 0.01, ^***^ *p* < 0.001, ^****^ *p* < 0.0001.

Next, the mechanisms by which fibrinogen deposition and accelerated MSU crystallization may worsen cartilage erosion in gout were explored, using intra‑articular injection of urokinase to achieve local FGG degradation. We first examined the expression levels of markers related to cartilage matrix‐degradation and injury response in FGG‐deposited regions [23, 24]. At the transcriptional level, compared with the injury+MSU group, *Comp*, *Anxa5*, and *Mmp13* gene expression levels were significantly decreased after intra‐articular urokinase injection (*p* < 0.05, *p* < 0.01, and *p* < 0.01, respectively), but remained higher than those in the injury group (Figure 6A). Immunofluorescence staining showed increased cathepsin K (CTSK) and tartrate‐resistant acid phosphatase (TRAP) protein signals in the injury+MSU group compared with the injury group (both *p* < 0.0001), whereas these signals were reduced after intra‐articular urokinase injection (*p* < 0.01, *p* < 0.0001, respectively; Figure 6B,C). Spatial co‐localization of fibrinogen, TRAP, and CTSK protein signals showed the same trend (Figure 6B,C). Repeating the experiments under uricase inhibition did not alter this trend (Figure S9). These findings support that injury‐induced fibrinogen deposition is accompanied by increased matrix‐degradation gene expression and stronger TRAP/CTSK‐positive resorption‐associated signals, whereas local urokinase treatment partially reduces these changes.

Human synovial single‐cell RNA sequencing (scRNA‐seq) data and plasma proteomic data were further integrated to validate these findings [25]. Synovial scRNA‐seq datasets from healthy donors, gout patients without cartilage injury, and gout patients with cartilage injury were further integrated, focusing on the mononuclear phagocyte lineage, including monocytes, macrophages, and osteoclasts (Figure 6D and Figure S10A). Synovial mononuclear phagocyte‐lineage cells from patients with gout with cartilage injury exhibited coordinated upregulation of integrin receptors, matrix metalloproteinases (MMPs), and bone‐resorption‐related genes compared to those with gout without cartilage injury (Figure S10B). Representative genes, including integrin subunit beta 5 (*ITGB5*), *MMP9*, and *CTSK*, presented the same pattern (Figure 6E). Enrichment analysis further supported a link between integrin‐related matrix responses and bone‐resorption/tissue‐degradation programs (Figure 6F). Pseudotime trajectory analysis revealed that, in gout patients with cartilage injury, mononuclear phagocyte lineage cells were positioned at a more terminal differentiation stage, with an increased shift toward matrix‐degrading phenotypes (Figure 6G,H). In the plasma proteomic data from gout patients in the UK Biobank (UKB) cohort, FGA protein levels were positively correlated with interleukin‐1 beta (IL‐1β), ITGA5, and MMP12 protein levels (Figure 6I), linking increased systemic fibrinogen to inflammatory activity, integrin‐related adhesion signaling, and matrix‐degrading activity. Together, the mouse experiments and human data analysis revealed the activation of integrin‐mediated matrix‐degrading programs at cartilage injury sites in gout, further supporting a “cartilage injury‐fibrinogen deposition‐accelerated MSU crystallization–cartilage erosion” self‐amplifying loop.

### 2.6 Urokinase Intervention or Repair Tissue Formation Could Alleviate MSU/fibrinogen‐Associated Cartilage Erosion

**FIGURE 7:**
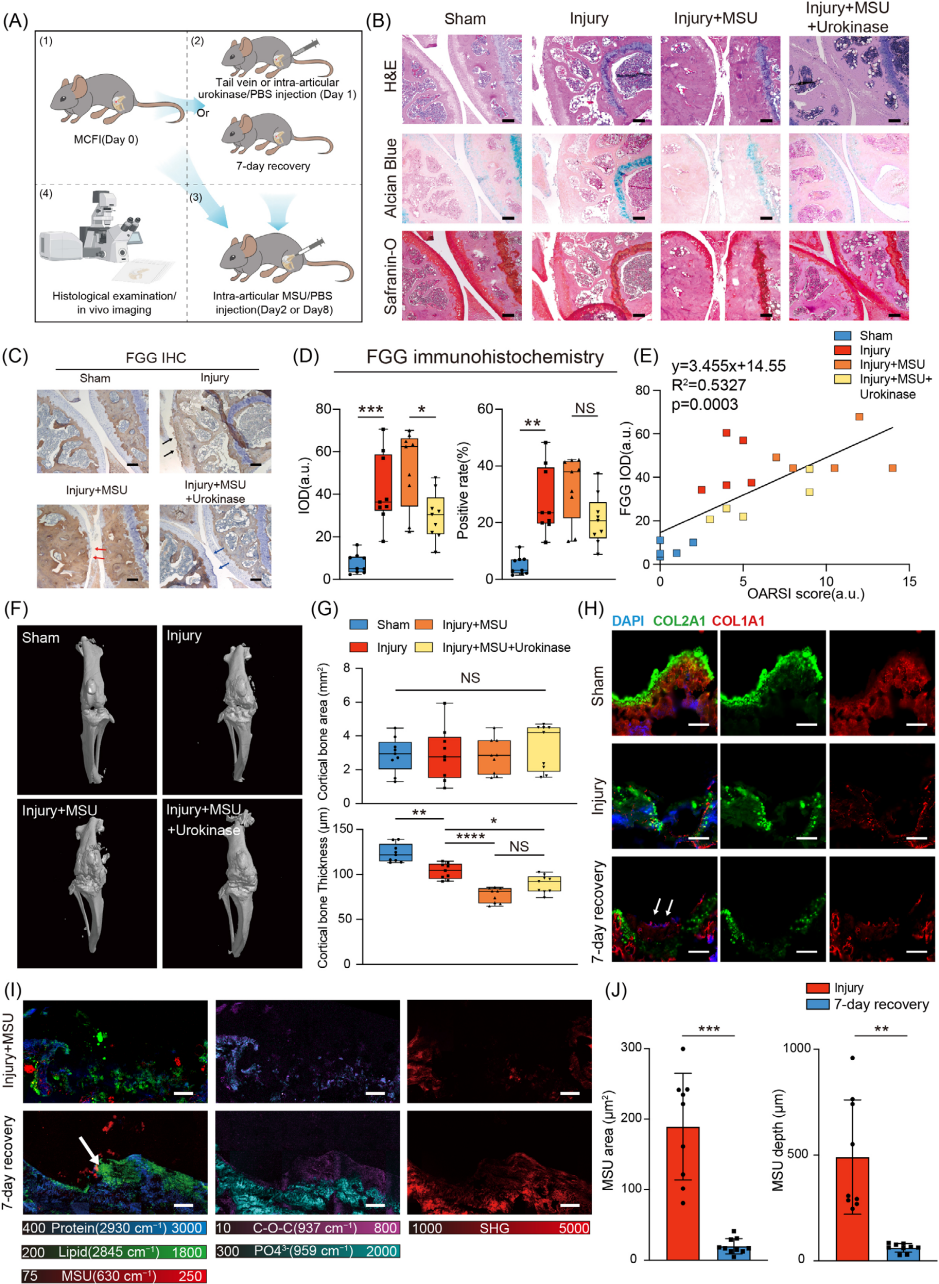
Urokinase intervention and fibrous repair tissue formation alleviate MSU‐associated cartilage erosion under fibrinogen deposition. (A) Schematic of the urokinase intervention and 7‐day recovery models used to reduce FGG protein deposition or limit deep MSU deposition after cartilage injury. (B) Histological staining showing cartilage matrix changes across the sham, injury, injury+MSU, and injury+MSU+urokinase groups, including hematoxylin and eosin, Alcian Blue, and Safranin O staining. (C) Immunohistochemistry staining showing FGG protein deposition in injured cartilage and adjacent regions across the indicated groups. Figure 7B to 7C show different staining methods for consecutive sections, allowing for the comparison of multiple staining results. (D) Immunohistochemical quantification showing reduced fibrinogen deposition after the intervention. (E) Correlation between FGG protein deposition and cartilage structural injury assessed by the Osteoarthritis Research Society International (OARSI) score. (F) Micro‐computed tomography (micro‐CT) images of mouse knee joints across groups. (G) Quantitative analysis of micro‐CT images, including cortical bone area and cortical bone thickness. (H) Immunofluorescence staining of collagen type I alpha 1 chain (COL1A1) and collagen type II alpha 1 chain (COL2A1) showing the collagen composition of fibrous repair tissue after the 7‐day recovery period. (I) Representative SRS images comparing the injury+MSU and 7‐day recovery groups across different SRS channels. For quantitative analysis, five mice were included per group (*n* = 5), and three representative fields of view were analyzed for each mouse. (J) Quantitative analysis of MSU deposition after fibrous repair tissue formation, including MSU deposition area and invasion depth. Scale bar: B, C, 50 µm; H and I, 100 µm. NS, not significant; ^*^ *p* < 0.05, ^**^ *p* < 0.01, ^***^ *p* < 0.001, ^****^ *p* < 0.0001.

Two intervention strategies were then designed to target this self‐amplifying loop: (1) reducing FGG protein deposition through urokinase treatment and (2) reducing deep MSU deposition by restoring the cartilage surface barrier through repair tissue formation after injury (Figure 7A) [26]. To determine whether targeting fibrinogen deposition could reduce MSU‐associated cartilage erosion, histological and in vivo imaging analyses were performed in the injury, injury+MSU, and injury+MSU+urokinase groups. Histological staining showed marked matrix disruption, proteoglycan loss, and disorganized cellular structure in the injury+MSU group; however, urokinase treatment reduced these pathological changes (Figure 7B). Immunohistochemical staining for FGG revealed that, in the injury+MSU+urokinase group, both FGG deposition at the damaged cartilage site and further crystal erosion were relieved, indicating that urokinase effectively reduces fibrinogen accumulation and alleviates the associated inflammatory response (Figure 7C). Subsequent quantitative analysis of FGG immunohistochemistry results (Figure 7D) showed that the IOD was significantly lower in the injury+MSU+urokinase group than in the injury+MSU group (*p* < 0.05). Moreover, cartilage structural injury was assessed using the Osteoarthritis Research Society International (OARSI) score. A significant positive correlation was observed between the OARSI score and FGG IOD (R2 = 0.5327, p = 0.0003; Figure 7E) [27]. Both the OARSI score and FGG IOD decreased after urokinase treatment (Figure 7E). Micro‐computed tomography (micro‐CT) of mouse knee joints presented surface irregularities in the injury group (Figure 7F, upper right). After MSU treatment, the joint surface became more irregular, with high‐density deposits attaching to the joint region, possibly related to urate crystal deposition (Figure 7F, lower left). However, micro‐CT showed fewer irregular deposits in the joint region and a smoother bone surface contour after urokinase treatment compared with the injury+MSU group (Figure 7F, lower right). The cortical bone area and thickness were further assessed in the quantitative analysis (Figure 7G). No significant difference in cortical bone area was observed among groups. However, the cortical bone thickness was significantly decreased after injury (*p* < 0.01). The cortical bone thickness was significantly higher in the urokinase intervention group than in the injury+MSU group (*p* < 0.05; Figure 7G). Together, these findings show that urokinase treatment reduced MSU‐associated cartilage erosion under fibrinogen deposition.

To determine whether post‐injury repair tissue formation could also break this self‐amplifying loop by limiting deep MSU deposition, a 7‐day recovery period was introduced between cartilage injury and intra‐articular MSU or phosphate‐buffered saline (PBS) injection. After the recovery period, fibrous repair tissue characterized by collagen type I was observed in the injured region, supporting the formation of a partial structural barrier on the injured cartilage surface (Figure 7H). Meanwhile, SRS imaging revealed that MSU signals were mainly restricted to the cartilage surface after fibrous repair tissue formation, with markedly reduced penetration into deeper tissues (Figure 7I). Quantitative analysis further showed that the MSU deposition area (*p* < 0.001) and invasion depth (*p* < 0.01) were significantly lower in the 7‐day recovery group (Figure 7J). These results indicate that breaking key steps in this self‐amplifying loop, either by reducing FGG protein deposition or by limiting MSU/fibrinogen deposition, can prevent further cartilage erosion in chronic gouty arthritis.

## 3 Discussion

Although studies have shown that cartilage injury or its matrix components can promote MSU crystal formation, the local factors and causal sequence linking sustained MSU deposition and progressive erosion remain unclear. Using in situ SRS imaging, the present study showed that injured cartilage is more susceptible to MSU‐associated erosion, with crystal deposition increasing in deeper regions in a crystallization‐frequency‐dependent manner. According to proteomic analysis, the enrichment of fibrinogen complex proteins was the most prominent change in the context of injured cartilage. Subsequently, a direct link was established between fibrinogen deposition and MSU crystallization: fibrinogen accelerated crystal formation, and FGG deposition co‐localized with deep MSU crystals. Mouse experiments and human data further connected fibrinogen deposition and accelerated MSU crystallization to integrin‐linked matrix‐degrading signaling. Together, these findings support a self‐amplifying “cartilage injury‐FGG deposition‐MSU crystallization‐further cartilage erosion” loop in chronic gouty arthritis. Moreover, interrupting this loop, either by reducing FGG deposition or restoring cartilage barrier, could attenuate further cartilage erosion.

Furthermore, an intact noncalcified cartilage layer was found to restrict MSU deposition, whereas its breach permitted deeper crystal invasion. More importantly, multicycle MSU crystallization not only increased crystal burden but also shifted crystal distribution toward deeper cartilage regions. Although Towiwat et al. used dual‐energy CT to demonstrate an “outside‐in” pattern of crystal deposition, their study's cross‐sectional nature limited further investigation into how crystals accumulate during this process and how crystals in different regions behave in gouty cartilage erosion [28]. The present study provides experimental evidence for this process in mouse cartilage by using ^14^N‐ and ^15^N‐labeled sodium urate in different crystallization cycles. More importantly, they highlight the importance of crystallization frequency, particularly the role of rapidly growing deep crystals, and offer a distinct perspective on the variability in the progression of cartilage erosion across individuals with gout. Specifically, patients with high flare frequency may be more susceptible to gout‐related cartilage injury than those with long disease duration but fewer flares [21, 29].

Another important finding is the enrichment of FGG at cartilage injury sites, which may play two previously unrecognized roles: promoting rapid MSU crystallization and integrin‐mediated matrix‐degrading programs at injury sites [30]. According to clinical studies, urate deposition is more common in joints with OA or early structural injury. The present data suggest that FGG converts structurally injured areas into crystal‐favorable microenvironments by accumulating at injury margins and deeper injured regions, thereby localizing and accelerating MSU deposition. Previous studies have also suggested that type II collagen can promote the occurrence of gout flares [7]. Nevertheless, the functional divergence of these two proteins suggests that they may influence gout through distinct mechanisms. Fibrinogen can quickly respond to local cartilage damage and inflammation, initiating and amplifying the erosion of cartilage in gout. While type II collagen is released into the joint after the initiation of cartilage destruction, it further accelerates the formation of MSU crystals and amplifies the integrin‐dependent macrophage recruitment process, thereby accelerating the progression of the disease.

This study also proposes some preliminary targeted strategies that may affect clinical practice after future refinement. Although adult articular cartilage has limited endogenous repair capacity, recent advances in cartilage tissue engineering, including scaffold‐mediated regeneration and biologics‐assisted repair, may provide potential approaches to preserve or restore the articular surface's structural integrity to stop the progress of erosion in gout patients [31]. In addition, intra‐articular fibrinolytic therapy may carry bleeding risks due to the rich blood supply of the synovium. Therefore, future studies should explore more locally selective strategies, including local delivery targeting fibrinogen deposition, nanoparticle‐mediated delivery, or low‐dose local fibrinolytic treatment, to limit MSU/fibrinogen deposition while reducing systemic side effects [32].

In summary, this study supports FGG protein deposition, FGG‐mediated local accelerated MSU crystallization, and activated integrin‐mediated matrix‐degrading programs as key components of a self‐amplifying loop that promotes cartilage erosion in chronic gouty arthritis. Breaking this loop, either by reducing FGG protein deposition through urokinase treatment or by reducing MSU deposition in deeper cartilage regions through restoration of the cartilage surface barrier after injury, reduces cartilage erosion. These findings provide new experimental evidence for understanding the progression of gout‐associated cartilage erosion and developing strategies that protect the cartilage barrier or target fibrinogen deposition.

## 4 Methods

### 4.1 Reagents

Information of reagents used in this study is listed in Table S2.

### 4.2 MSU Crystallization Assay

Crystallization was performed using previously described methods [33] with modifications (Figure 2A). Uric acid (250 mg/dL) was dissolved in DMEM at 37°C and then added with sodium hydroxide (175 mg/dL). The pH of the solution was adjusted to 7.4 using diluted hydrochloric acid. Then, the resulting saturated urate solution was used for tissue explant crystallization assays or injected intra‐articularly for in vivo experiments.

To co‐crystallize fibrinogen with MSU, fibrinogen (10 mg/mL) was dissolved in saline as the stock solution. Subsequently, it was added to the system at a final concentration of 2 or 4 mg/mL before the addition of hydrochloric acid. Each system was divided into five aliquots and crystallized for 1, 2, 4, 12, or 24 h. Thereafter, the MSU crystals were filtered and dried to measure the crystal mass in accordance with a previous study [34]. As a control, parallel crystallization reactions were conducted under identical conditions, without the addition of fibrinogen.

For all crystallization experiments, aliquots of the crystallization solutions were collected, and their endotoxin‐free status was confirmed using the E‐Toxate Reagent.

### 4.3 Mouse Model

Animal procedures were performed under the approved protocol described in the Ethics Approval Statement. Male C57BL/6 mice (8 weeks old, Shanghai Laboratory Animal Research Center, Shanghai, China) were used in this study. The medial condylar femoral injury (MCFI) model and medial meniscus destabilization model for OA (*n* = 5 per group) were used as joint injury models [35, 36]. Anesthesia was induced with 3% isoflurane and maintained with 1.5% isoflurane. On day 0, mice underwent MCFI surgery. MSU injections were performed on day 2 after injury (early phase), and animals were sacrificed 24 h later for histological analysis.

For the urokinase intervention model, mice received either systemic urokinase treatment via tail vein injection (1 mg/kg; Aladdin, U108373) or local urokinase treatment via intra‐articular injection into the knee joint (50 µg/mL, 20 µL per knee; final dose, 1 µg per knee, 120 IU per knee) on day 1. PBS was administered using the same route as the corresponding control treatment. For the 7‐day recovery model, MSU (50 mg/mL) injection and final tissue collection were delayed by 7 days, allowing us to determine whether fibrous cartilage repair tissue helps break the pathological progression of gouty cartilage erosion.

Mice received daily intraperitoneal injections of potassium oxonate (300 mg/kg/day; Aladdin) or saline for 7 days before MCFI and intra‐articular MSU injection to exclude the possibility of species differences (the lack of uricase in humans) affecting the main conclusions of this study. Cartilage was collected 24 h later to assess MSU deposition and joint pathology.

### 4.4 Isotope Labeling and Crystallization Cycles

Isotopic labeling was used to distinguish MSU crystals that were formed during the sequential crystallization cycles. In the first cycle, cartilage explants were incubated in a saturated ^14^N‐labeled sodium urate solution in DMEM at 37°C for 24 h. After rinsing with pre‐warmed PBS, the explants were transferred to a freshly prepared saturated 1,3‐^15^N_2_‐labeled sodium urate solution for an additional 24‐h incubation (second cycle). No intermediate incubation in a non‐saturated solution was included between cycles. The resulting isotopically labeled crystals were imaged and analyzed using SRS microscopy. Raman spectral peaks near 1210 and 1196 cm^−1^ corresponding to ^14^N‐ and ^15^N‐labeled MSU crystals, respectively, were used to distinguish crystals from different cycles.

### 4.5 Cartilage Explant Culture

The explant culture methods were adapted from a previous study [13]. Briefly, explants were cultured with DMEM (high glucose, Thermo Fisher Scientific 11965092) in six‐well plates at 37°C and 5% CO_2_ supplemented with antibiotics (penicillin‐streptomycin 10 000 U/mL, Gibco 15140122).

### 4.6 Histology

Mouse synovial tissue was stained and analyzed as described in a previous study [23]. Cartilage matrix‐degradation was analyzed via Safranin O‐fast green and Alcian blue staining. Meanwhile, fibrinogen deposition was assessed using immunohistochemistry. Fibrinogen was quantified using ImageJ, and the positive area and IOD were analyzed [7]. Data were obtained from five mice per group, using three cartilage sections per mouse.

Immunofluorescence staining of mouse knee joint samples was also performed as described in a previous study [25]. The antibody information is listed in Table S3. The positive signal area, fluorescence intensity, and spatial co‐localization between different markers were quantified using ImageJ as described in a previous study [25]. Data were obtained from six mice per group, using three representative joint sections per mouse. Femoral bone injury was evaluated using the OARSI scoring system [27]. Scoring was performed by independent observers who were blinded to the experimental groups.

### 4.7 Quantitative Reverse Transcription Polymerase Chain Reaction

Quantitative reverse transcription polymerase chain reaction was performed as described in a previous study [7]. Detailed information on reagents, instruments, and primer sequences is provided in Table S4.

### 4.8 ELISA

Serum fibrinogen levels were measured using an ELISA kit in accordance with the manufacturer's instructions. The measurements were performed in duplicate.

### 4.9 Micro‐CT

Mouse knee joint bone tissue was extracted and placed on a micro‐CT sample stage (SkyScan 1076 micro‐CT, Bruker). Data were reconstructed using DataViewer, and the resulting images were visualized using CTAn software.

### 4.10 5D Label‐Free Mass Spectrometry

Cartilage samples were collected from the intercondylar notch region of mouse knee joints (sham, injury, MSU, and injury+MSU groups; *n* = 3 per group). The tissues were flash‐frozen, pulverized in liquid nitrogen, and precipitated in trichloroacetic acid/acetone (1:9) at −20°C for 4 h. After centrifugation and acetone washing, the protein pellets were solubilized in SDT buffer (4% SDS, 100 mm Tris‐HCl, pH 7.6), boiled, sonicated, and cleared through centrifugation. The protein concentrations were determined using a bicinchoninic acid protein assay kit (Beyotime).

For liquid chromatography‐tandem mass spectrometry, the proteins were reduced with dithiothreitol, alkylated with iodoacetamide, and digested overnight with trypsin (1:50, w/w). The peptides were desalted on C18 columns and quantified by ultraviolet absorbance at 280 nm. Samples were analyzed on the timsTOF Pro Mass Spectrometer (Bruker, Bremen, Germany) operated in the PASEF mode, and peptide identification was performed using PaSER 2023 software.

### 4.11 Spontaneous Raman Spectroscopy

Spontaneous Raman scattering spectra were acquired using a home‐built Raman spectrometer, including a monochromator (iHR320, Horiba), charge‐coupled device camera (Symphony, Horiba), and microscope frame (IX71, Olympus) with a 40× air objective with a 633 nm helium‐neon laser beam at room temperature. Raman spectra were acquired at 10 s per spectrum, averaged 10 times using the same conditions for all samples. A quartz plate was used to suppress the autofluorescence generated by the glass slide.

### 4.12 SRS Microscopy

SRS imaging was performed using a home‐built laser scanning microscope based on a commercial femtosecond laser system (Insight DS+, Spectra‐Physics). The Stokes beam (1040 nm, ∼200 fs) and tunable pump beam (680–1300 nm, ∼150 fs) were synchronized at 80 MHz. Both beams were temporally stretched (pump: ∼3.8 ps, Stokes: ∼1.8 ps) via SF57 glass rods to achieve a spectral resolution of ∼13 cm^−1^. The Stokes beam was modulated at 20 MHz using an electro‐optical modulator (Thorlabs), and both beams were spatially and temporally overlapped via a dichroic mirror before being directed into a galvanometer‐based laser scanning unit (FV1200, Olympus).

Samples were imaged using water‐immersion objectives (25× NA 1.05 for tissue; 60× NA 1.2 for crystal details). Forward‐scattered signals were collected through a high‐NA condenser (NA = 1.4, Nikon), filtered (CARS ET890/220, Chroma), and detected by a custom photodiode. The signals were demodulated by a lock‐in amplifier (HF2LI, Zurich Instruments) and processed for image formation. The Raman shifts at 2845 and 2930 cm^−1^ (lipids/proteins) were excited using an 802‐nm pump. Meanwhile, a 920‐nm pump was used for signals from isotopically labeled MSU crystals (∼1200 cm^−1^). SHG signals for collagen were simultaneously collected in the epi‐mode using a 405/10‐nm filter and photomultiplier tube. All images were acquired at 512 × 512 pixels with 2 µs dwell time and ∼350 nm lateral and ∼1 µm axial resolution. Mosaicking was applied for large‐area tissue reconstruction. The laser powers at the sample were 30 mW (pump) and 40 mW (Stokes). SRS‐imaged and non‐imaged control sections were compared after histological staining to control for potential imaging‐induced tissue effects. The imaging‐control experiment was performed under the same laser‐power and exposure conditions used in this study.

### 4.13 Image Processing

Image processing was performed using ImageJ software as described in a previous study [7, 25]. For two‐component unmixing, spectral decomposition was used for lipid (2845 cm^−1^)/protein (2930 cm^−1^) decomposition and PO_4_^3−^ (959 cm^−1^)/C─O─C (938 cm^−1^) decomposition in tissues [17].

For N^14^‐ and N^15^‐labeled MSU crystal quantification, the spatial distribution maps of N^14^‐ and N^15^‐labeled MSU crystals were first reconstructed using an MCR algorithm [37].

### 4.14 Definition and Segmentation of the Tidemark and Cement Line in SRS Images

For SRS‐based osteochondral boundary annotation, the tidemark was defined as the upper interface of the PO_4_^3−^ signal‐positive calcified cartilage region separating C─O─C‐rich noncalcified cartilage from calcified cartilage. By contrast, the cement line was defined as the lower interface of the compact calcified cartilage plate, where the continuous PO_4_^3−^ signal transitions to the discontinuous/trabecular subchondral bone pattern. Co‐registered C─O─C (938 cm^−1^) and PO_4_^3−^ (959 cm^−1^) SRS images were percentile‐normalized and Gaussian‐smoothed. The PO_4_^3−^ channel was binarized using Otsu's threshold to extract the tidemark as the first PO_4_^3−^ signal‐positive pixel along each image column. For cement line detection, the C─O─C channel was contrast‐enhanced and combined with a local PO_4_^3−^ continuity map, and the algorithm searched beneath the tidemark for the strongest joint boundary cue (defined by the loss of compact PO_4_^3−^ continuity together with a C─O─C contrast transition). In severely eroded regions, missing boundary segments were reconstructed from adjacent intact anchors through cubic Hermite interpolation, and all detected/reconstructed lines were visually reviewed on merged C─O─C/PO_4_^3−^ images. The analysis code is available at: https://github.com/XiaofengZhou16/srs‐osteochondral‐boundary‐segmentation.

### 4.15 scRNA‐seq Data Integration and Preprocessing

Synovial tissue scRNA‐seq data were obtained from our previously published work [25]. The original experimental protocols involving human specimens were approved by the Ethics Committee of Huashan Hospital (KY2020‐060). Written informed consent was obtained from all participants.

Patients were then reclassified based on surgical records into two groups: gout patients with or without cartilage injury. Then, quality control filters were applied to exclude low‐quality cells. Specifically, cells that met any of the following criteria were removed: (1) total unique molecular identifier counts <500 or >60 000, (2) number of expressed genes <200 or >7500, or (3) mitochondrial gene content >20% or ribosomal gene content >50% (Figure S10C). Following quality control, dataset integration, dimensional reduction, cell clustering, and cell type annotation were performed as previously described (Figure S10D,E and Table S5) [25].

### 4.16 Differential Gene Expression and Enrichment Analysis

Differential gene expression analysis was conducted as previously described to characterize transcriptional differences among mononuclear phagocyte populations under different conditions [27]. Genes with |log_2_ fold change| > 1 and adjusted p < 0.05 were considered significant. The detailed results are described in Table S6.

Significantly upregulated genes were further analyzed using Kyoto Encyclopedia of Genes and Genomes pathway enrichment analysis as described in a previous study [25].

### 4.17 Pseudotime Trajectory Analysis

Pseudotime trajectory analysis was performed as described in a previous study [25]. A matrix‐degradation‐associated signature was calculated using the predefined gene set listed in Table S7. Genes available in the Monocle2 dataset were retained. Expression values were normalized by cell‐specific size factors, log2‐transformed after adding a pseudo‐count of 1, scaled for each gene, and averaged across the available signature genes to obtain a cell‐level signature score. The signature score was then plotted along Monocle2 pseudotime. Group differences were tested using the Wilcoxon rank‐sum test or Kruskal‐Wallis test, depending on the number of groups.

### 4.18 Study Population of UKB Data Analysis

This research was conducted under approved UKB application number 742419 [38]. At baseline, the participants completed touchscreen questionnaires, underwent physical measurements, and provided biological samples. The participants included in this study underwent plasma proteomic profiling at baseline and subsequently developed incident gout during follow‐up. Individuals were required to have complete data for all key covariates. The final analytic sample comprised 1197 participants who met these inclusion criteria.

### 4.19 Blood Proteomics

Plasma proteomics were examined and quantified through the Olink Explore Proximity Extension Assay platform, which measured 2923 unique proteins. Normalized Protein expression (NPX) values were generated following quality control, normalization, and batch effect correction [39, 40]. The details of proteins on the Olink Explore 3072 platform are described in Table S8.

### 4.20 Outcome Definitions

The primary outcome was incident gout, defined by the International Classification of Diseases, Tenth Revision code M10. Incident cases were identified as gout diagnoses occurring after the baseline visit. The event date corresponded to the earliest available record across multiple data sources (i.e., first occurrence, death registries, or hospital inpatient records), whichever came first. The definitions for the disease phenotype are described in Table S9. Moreover, detailed field descriptions are available on the UK Biobank showcase website.

### 4.21 Correlation Analysis Between FGA and Other Proteins

Correlation analysis was performed with a predefined panel of proteins implicated in cartilage matrix‐degradation, extracellular matrix remodeling, and inflammatory signaling to investigate molecular associations with FGA expression. Pairwise protein correlations were computed using Spearman's rank correlation coefficient (ρ) to account for potential non‐normality in protein distributions. The ρ values and corresponding two‐sided p‐values were reported. Detailed information about the predefined panel of proteins and analysis results are described in Table S10.

### 4.22 Statistical Analysis

Statistical analyses were performed using GraphPad Prism v8.0.1 (GraphPad Software, La Jolla, CA, USA). Data normality was assessed using the Shapiro‐Wilk test. For two‐group comparisons, unpaired two‐tailed *t*‐tests were used for normally distributed data, and the Mann‐Whitney U tests were used for non‐normally distributed data. For multiple group comparisons, one‐way analysis of variance with Tukey's post hoc test or Kruskal‐Wallis with Dunn's test was applied based on data distribution.

The results are reported as the mean ± standard error of the mean for normally distributed data or the median with interquartile range for non‐normally distributed data. All statistical tests were two‐tailed, and statistical significance was considered at *p* < 0.05. Exact *n* values (biological replicates) and p‐values are provided in the figure legends. For immunohistochemistry, three technical replicates per sample were averaged. The representative images or spectra reflect consistent results from at least three independent experiments.

## Author Contributions

Y.H. and M.J. were responsible for the overall content as the guarantor. Y.H., M.J., X.H., and N.D. supervised and designed the study and also edited the manuscript. X.H. and S.B. prepared the samples and performed stimulated Raman scattering microscopy experiments. X.H. and Z.L. performed molecular biology experiments and wrote the manuscript. Z.L. and X.Z. performed bioinformatic analysis for human transitional section. H.Q. performed preliminary experiments of MSU crystallization.

## Funding

This work is supported by National Key R&D Program of China (2021YFF0502900, to M.J.); Natural Science Foundation of Shanghai (25ZR1402050, to H.X.); Shanghai Magnolia Program Pujiang Project (24PJD009, to H.X.); Shanghai Post‐doctoral Excellence Program (2024097, to H.X.); Postdoctoral Fellowship Program of CPSF (GZC20240290, to H.X.); China Postdoctoral Science Foundation (2025M772323, to H.X.); National Natural Science Foundation of China (62425501, to M.J.).

## Ethics Statement

All animal experiments were approved by the Animal Welfare and Ethics Group at the Department of Experimental Animal Science, Shanghai Medical College of Fudan University (approval number: 2019020405) and performed based on the ARRIVE guidelines (https://arriveguidelines.org). Human tissue scRNA‐seq data were obtained from our previously published work [25]. The original experimental protocols involving human specimens were approved by the Ethics Committee of Huashan Hospital (KY2020‐060). Written informed consent was obtained from all participants.

## Consent

All participants provided written informed consent.

## Conflicts of Interest

Nicola Dalbeth has received consulting fees, speaker fees or grants from Novartis, Horizon, Selecta, Arthrosi, JW Pharmaceutical Corporation, PK Med, LG Chem, JPI, PTC Therapeutics, Protalix, Unlocked Labs, Hikma, Dexcel Pharma, Shanton Pharma, Sobi, Avalo, Biomarin, Crystalys, Medcryst outside the submitted work. Nicola Dalbeth is supported by the Health Research Council of New Zealand.

## Supporting information




**Supporting File 1**: advs76383‐sup‐0001‐SupMat.docx.

**Supporting File 2**: advs76383‐sup‐0002‐TableS1.xlsx.

**Supporting File 3**: advs76383‐sup‐0003‐TableS2.xlsx.

**Supporting File 4**: advs76383‐sup‐0004‐TableS3.xlsx.

**Supporting File 5**: advs76383‐sup‐0005‐TableS4.xlsx.

**Supporting File 6**: advs76383‐sup‐0006‐TableS5.xlsx.

**Supporting File 7**: advs76383‐sup‐0007‐TableS6.xlsx.

**Supporting File 8**: advs76383‐sup‐0008‐TableS7.xlsx.

**Supporting File 9**: advs76383‐sup‐0009‐TableS8.xlsx.

**Supporting File 10**: advs76383‐sup‐0010‐TableS9.xlsx.

**Supporting File 11**: advs76383‐sup‐0011‐TableS10.xlsx.

## Data Availability

The data that supports the findings of this study are available in the supplementary material of this article. The data from the UK Biobank can be requested through the standard protocol (https://www.ukbiobank.ac.uk/register‐apply/).
