# Supplementary material for: Identification of an Osteochondral Erosion Process in Gout Driven by Fibrinogen‐Integrin Activation and Local MSU Crystallization
Source: Adv Sci (Weinh). 2026 Jul 13:e76383. Online ahead of print. doi: 10.1002/advs.76383 (PMC13360169; doi:10.1002/advs.76383)
Supplement: Supplementary file 1 — Supporting File 1: advs76383‐sup‐0001‐SupMat.docx. [file ADVS-9999-e76383-s011.docx]

Supplementary Materials

**
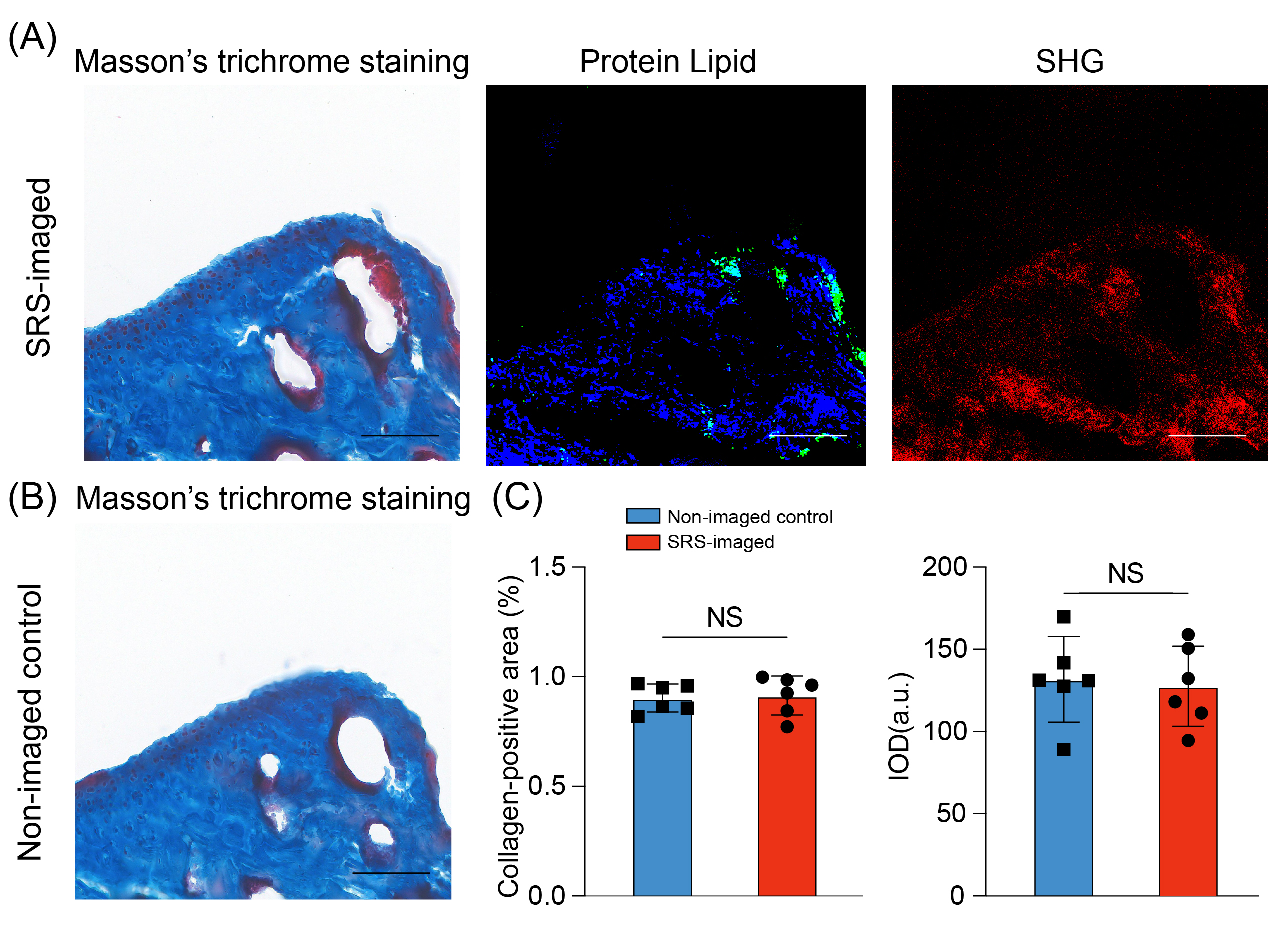
Figure S1. Evaluation of potential SRS imaging-induced effects on cartilage tissue.
(A-B)** Representative Masson’s trichrome staining images of adjacent cartilage sections from the SRS-imaged group and non-imaged control group. Scale bars: 100 μm. **(C)** Quantitative analysis of the collagen-positive area ratio and integrated optical density (IOD). For quantitative analysis, 6 mice were included per group (n = 6), and 1 representative fields of view were analyzed for each mouse. NS, not significant.


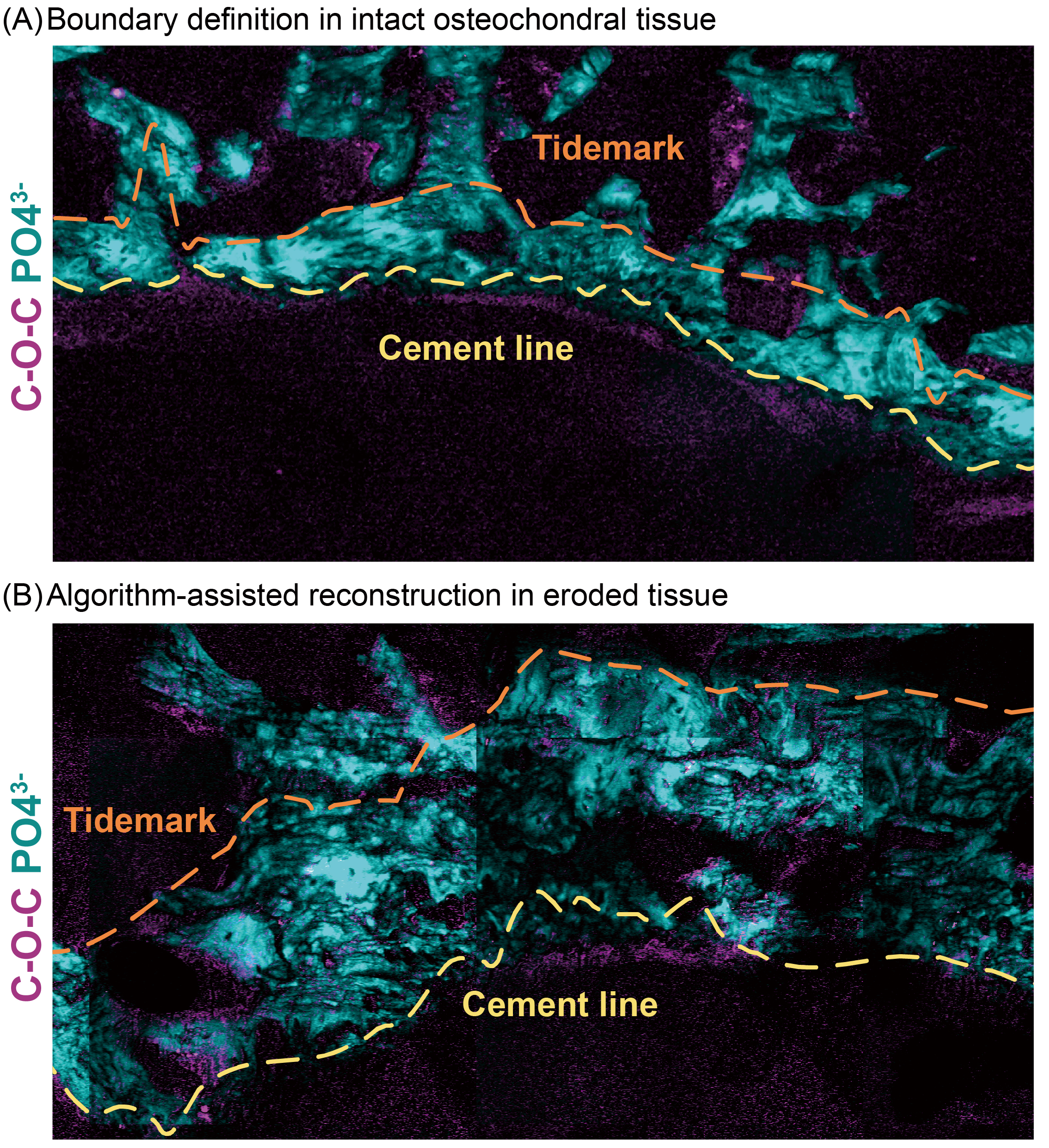
**Figure S2. Algorithm-assisted definition and reconstruction of the tidemark and cement line in SRS images.** **(A)** Merged C-O-C (magenta, 938 cm⁻¹) and PO₄³⁻ (cyan, 959 cm⁻¹) SRS image from an intact osteochondral section showing the anatomical definitions of the tidemark and cement line. The tidemark was defined as the upper interface of the PO₄³⁻-positive calcified cartilage region separating noncalcified cartilage from calcified cartilage. The cement line was defined as the lower interface of the compact calcified cartilage plate separating calcified cartilage from subchondral bone. **(B)** Representative eroded osteochondral section showing algorithm-assisted boundary assignment and reconstruction in regions with disrupted tissue architecture. Missing boundary segments were reconstructed from adjacent intact anchors using cubic Hermite interpolation and then visually reviewed on merged C-O-C/PO₄³⁻ SRS images. Orange lines indicate the tidemark, and yellow dashed lines indicate the cement line.

**
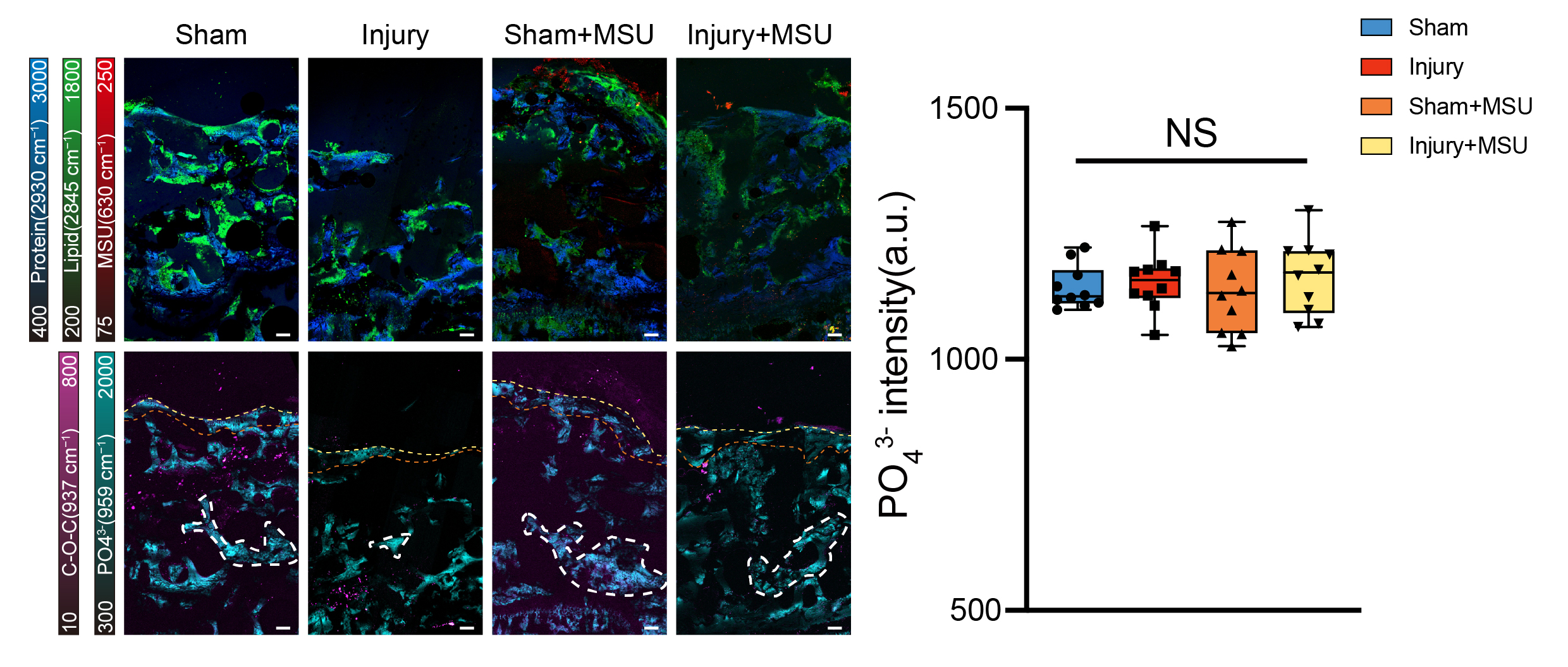
Figure S3. SRS imaging and quantification of cartilage composition.** Left: Representative SRS images of the sham, injury, sham+MSU, and injury+MSU groups in the same experimental dataset. Yellow dashed lines, orange dashed lines, and the white dashed area indicate the tidemark, cement line, and the subchondral bone plate, respectively. Scale bars, 100 μm. Right: Quantification of PO₄³⁻ intensity showed no significant differences (p > 0.05). n = 10 fields per group.

**
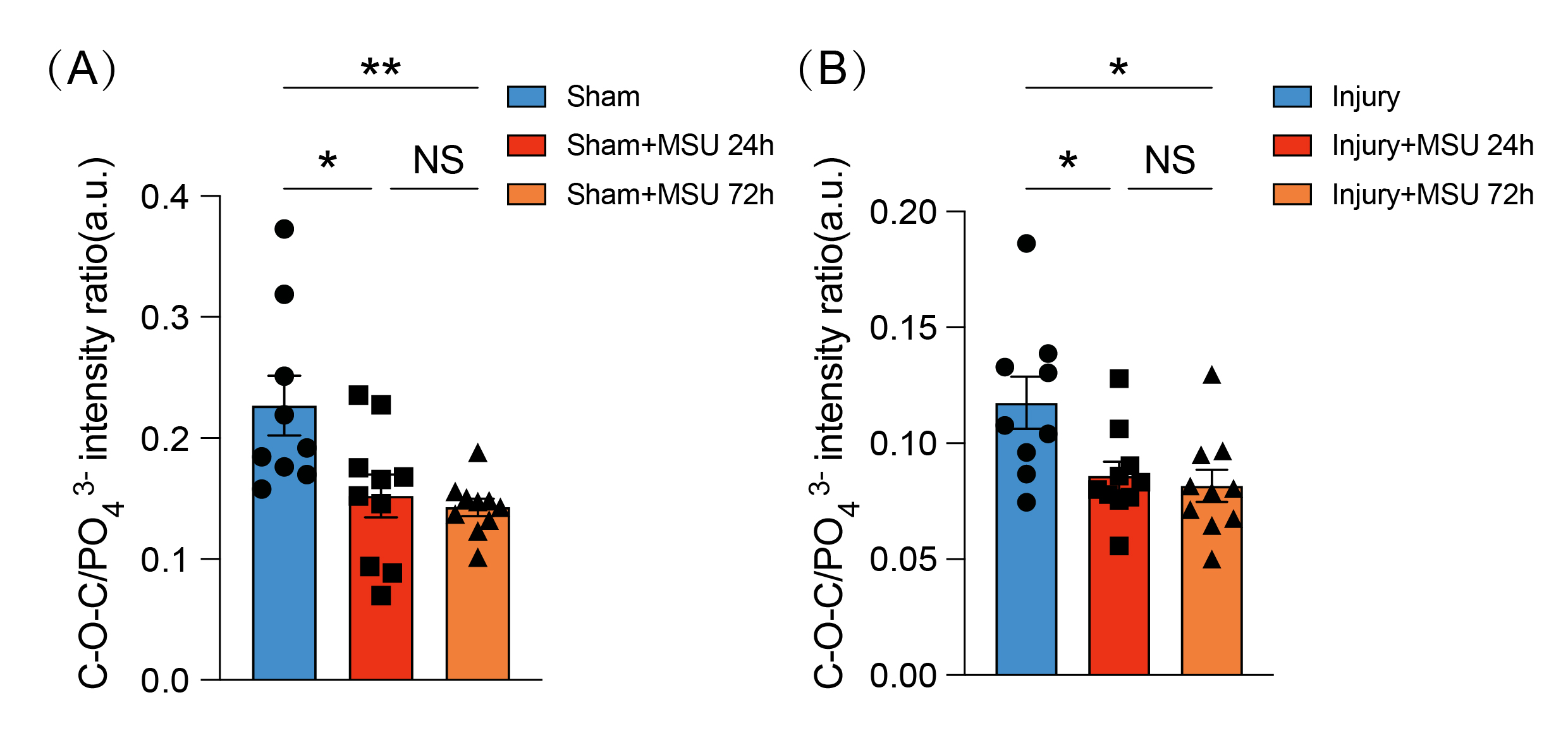
Figure S4. Quantitative analysis of the C-O-C/PO_4_^3-^ ratio after MSU crystallization in sham and injury group. (A)** Quantitative analysis, normalized to the signal intensity at 959 cm^−1^, showing the C-O-C/PO_4_^3-^ intensity ratio，presenting matrix changes after 24 hours, 72 hours MSU crystallization in sham group. **(B)** Quantitative analysis, normalized to the signal intensity at 959 cm^−1^, showing the C-O-C/PO_4_^3-^ intensity ratio，presenting matrix changes after 24 hours, 72 hours MSU crystallization in injury group. For quantitative analysis, 3 mice were included per group (n = 3), and 2-3 representative fields of view were analyzed for each mouse. Data are presented as mean ± SEM. NS, not significant; P < 0.05, **P < 0.01, ***P < 0.001, ****P < 0.0001.

**
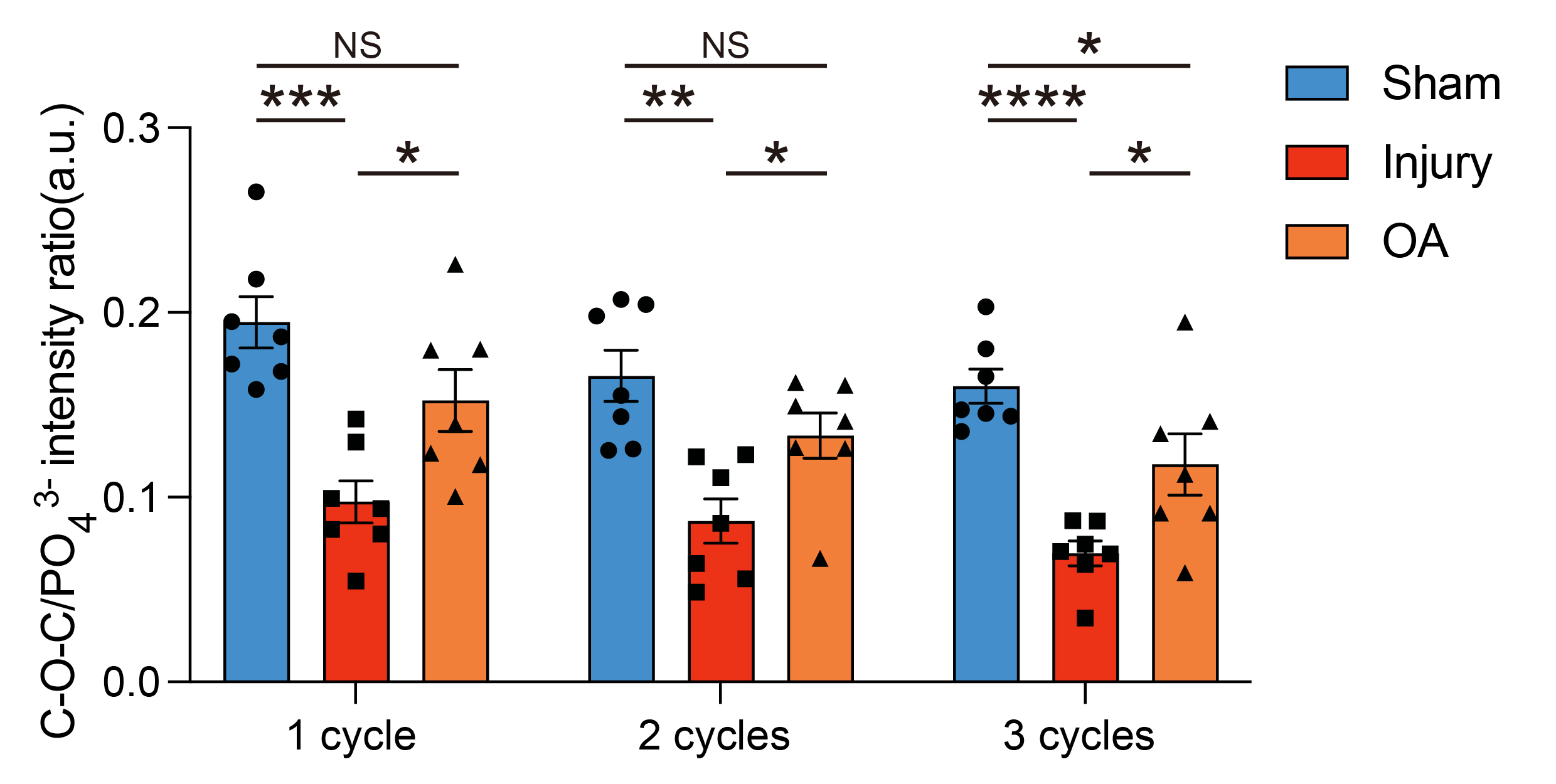
Figure S5.** The C-O-C/PO43- intensity ratio in sham, injury, and OA cartilage explants after one, two, or three MSU crystallization cycles. Data are presented as mean ± SEM (7 fields per gourp per cycle). NS, not significant; P < 0.05, **P < 0.01, ***P < 0.001, ****P < 0.0001.

**
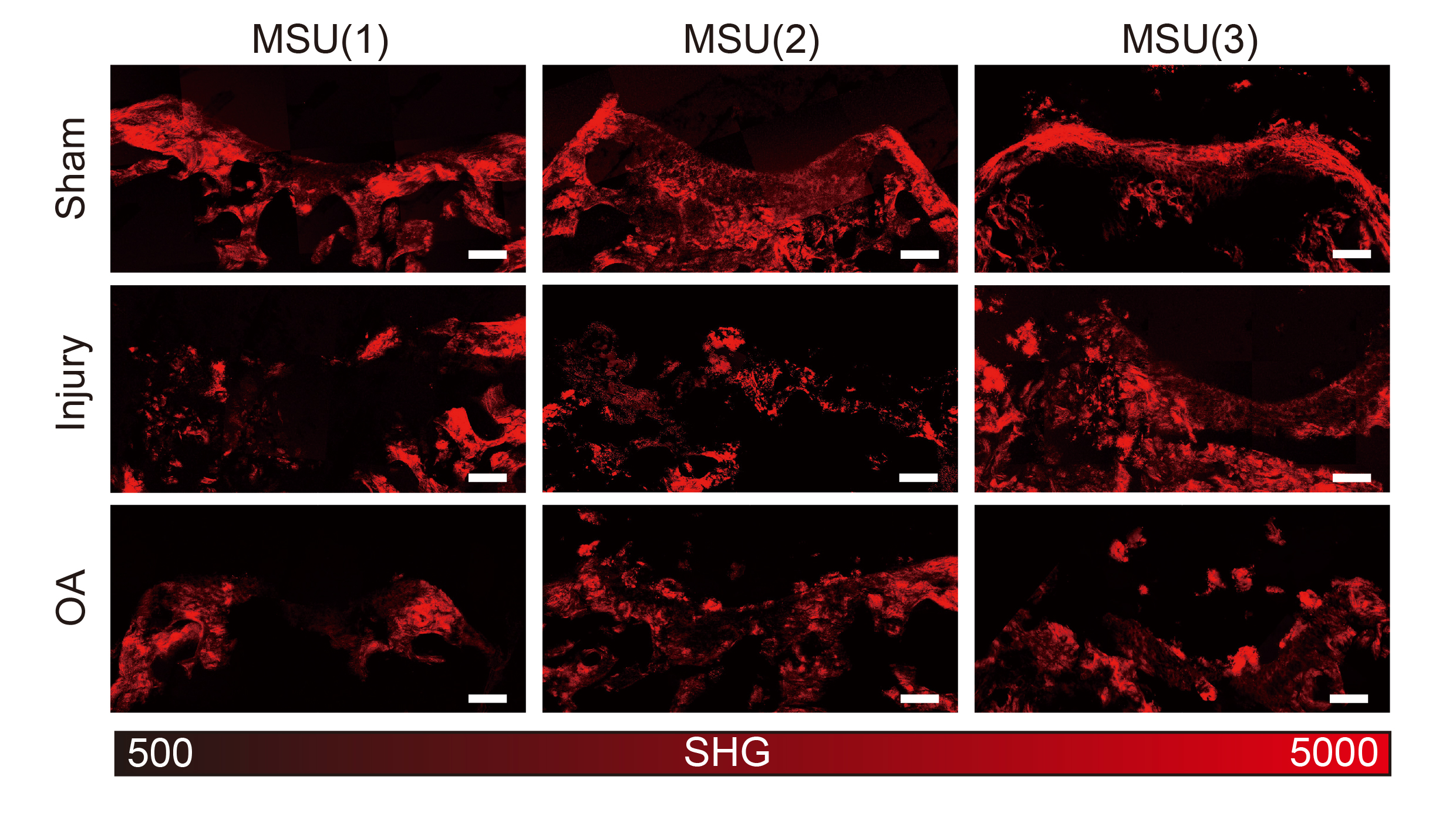
Figure S6** Representative images from the normal, OA, and injury groups co-cultured with MSU for 1–3 cycles across different SHG channels with signal ranges standardized using a look-up table. Scale bar: 100 µm.

**
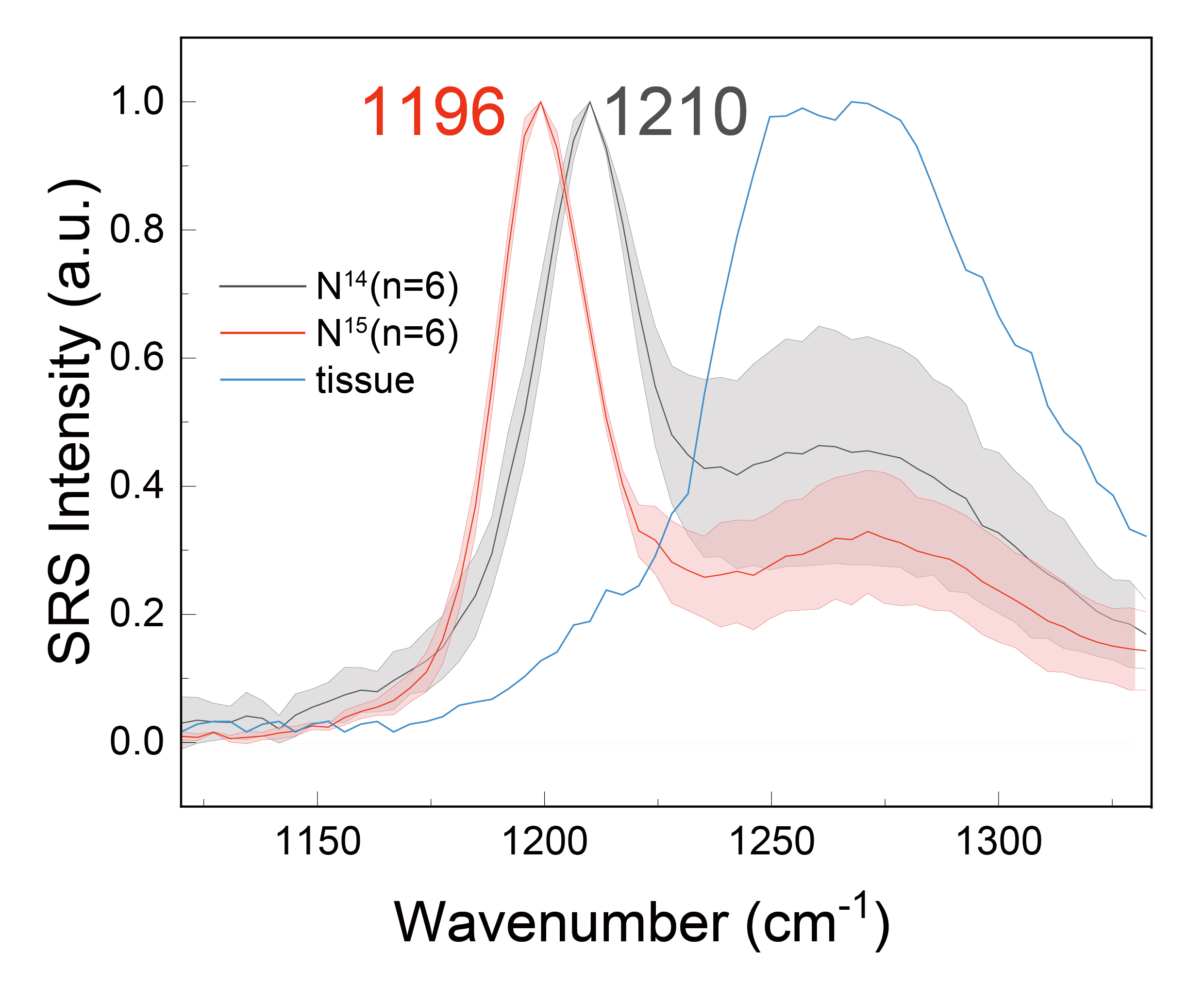
Figure S7.** SRS spectra of MSU crystals at Raman peak of 1210 cm-1 (gray) made from the crystallization of N^14^-labeled uric acid, MSU crystals at Raman peak of 1196 cm-1 (red) made from the crystallization of N^15^-labeled uric acid and cartilage tissue (blue), respectively.

**
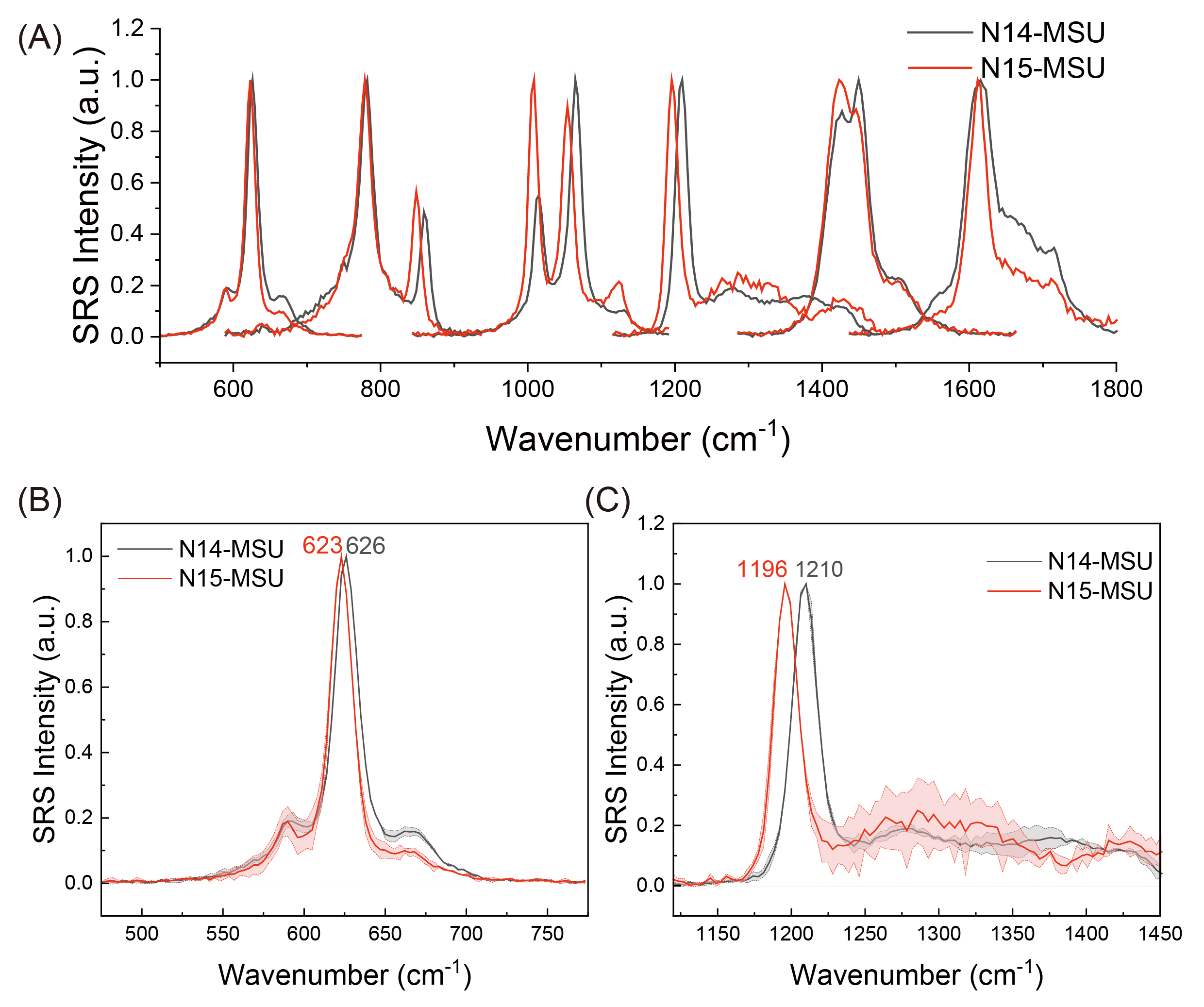
Figure S8.** SRS spectra of MSU crystals. (A) SRS spectra of N^14^-labeled and N^15^-labeled MSU crystals at different Raman range. (B) and (C) SRS spectra of N14-labeled and N^15^-labeled MSU crystals at Raman peak of ~626 cm^-1^ and ~1200 cm^-1^, respectively. The Raman peak of N^15^-labeled MSU crystals has a larger redshift around 1200 cm^-1^.

**
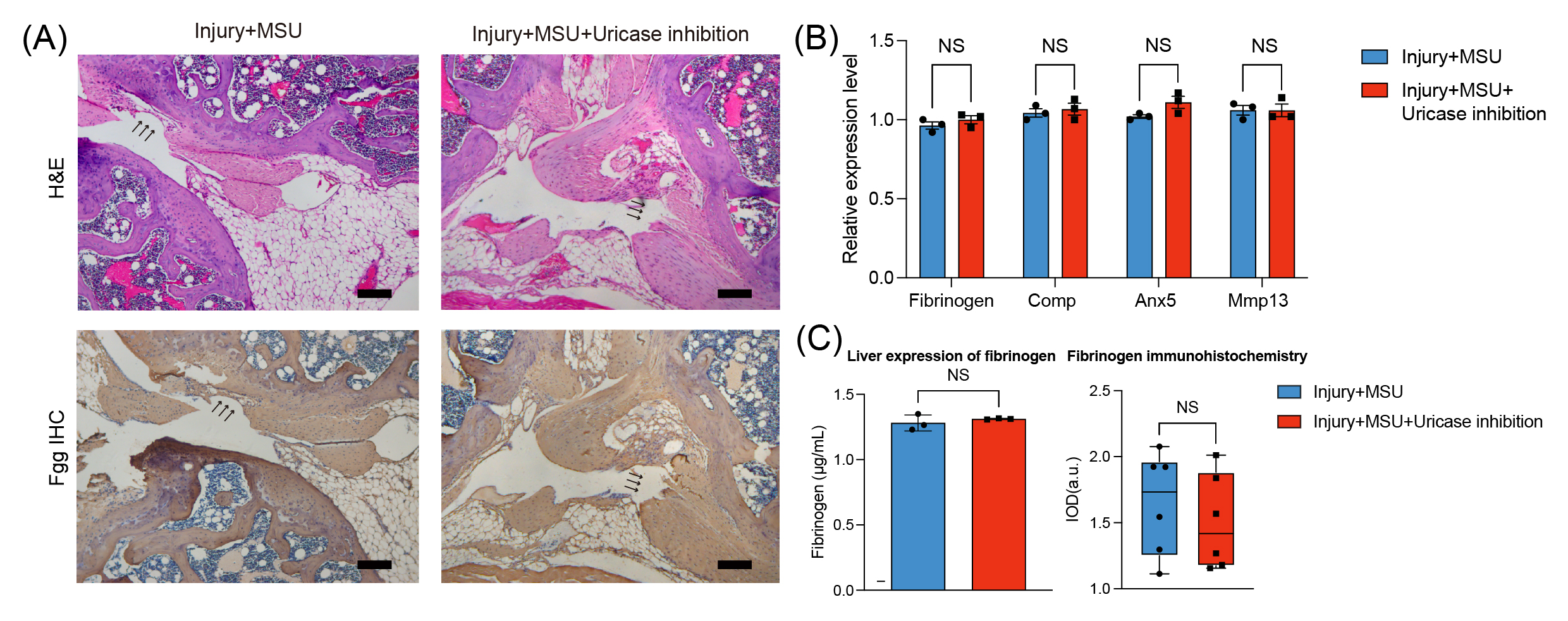
Figure S9.** Effects of uricase inhibition on fibrinogen expression and cartilage matrix components in injured joints. (A) Representative H&E (top) and fibrinogen gamma chain (Fgg) immunohistochemistry (bottom) staining of knee joints from Injury+MSU and Injury+MSU+Uricase inhibition groups. Scale bars, 100 μm. (B) Quantitative RT-PCR analysis of cartilage matrix-related genes (*Fibrinogen*, *Comp*, *Anx5*, and *Mmp13*) in cartilage tissue (n = 3 per group). (C) Left: Plasma fibrinogen concentration measured by ELISA (n = 3 per group). Right: Quantification of Fgg immunohistochemical staining intensity (IOD) in cartilage sections. For quantitative analysis, 3 mice were included per group (n = 3), and 2 representative fields of view were analyzed for each mouse. NS, not significant; P < 0.05, **P < 0.01, ***P < 0.001, ****P < 0.0001.

**
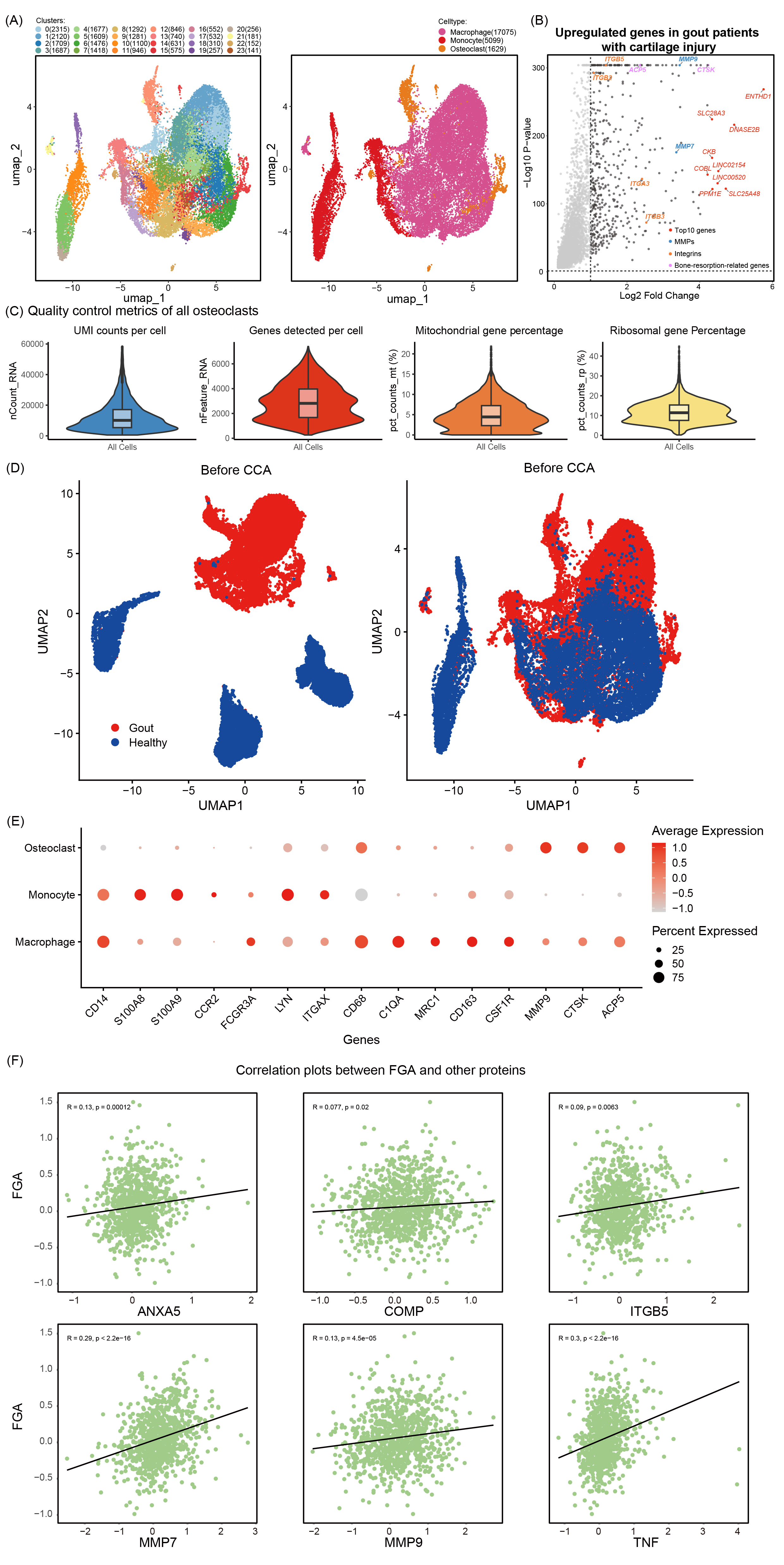
**

**Figure S10. scRNA-seq and UK Biobank analysis of fibrinogen-integrin signatures.** **(A)** UMAP plots of integrated synovial mononuclear cell data visualized by cell clusters (Left) and celltype (Right). **(B)** Volcano plot showing upregulated genes in synovial mononuclear cells from gout patients with cartilage injury versus those without cartilage injury. Top 10 upregulated genes (red), matrix-degrading enzyme genes (blue), integrin family genes (orange), and bone-resorption-related genes (purple) are highlighted. **(C)** Violin plots showing quality control metrics of integrated synovial mononuclear cell data, including UMI counts, gene numbers, mitochondrial gene percentage, and ribosomal gene percentage. **(D)** UMAP plots of synovial mononuclear cells from gout and healthy donors before and after Canonical Correlation Analysis (CCA) integration. **(E)** Dot plot showing marker gene expression across annotated osteoclasts, monocytes, and macrophages from synovial mononuclear cell data. **(F)** Correlation plots showing associations between plasma FGA levels and selected plasma proteins in gout patients.
